# Bugs and germs in GI‐disease

**DOI:** 10.1002/ueg2.12693

**Published:** 2024-11-13

**Bugs and germs in GI‐disease: Late‐breaking abstracts**

**17:00–18:00/A3**

## LB01 | TARGETED FECAL MICROBIOTA TRANSPLANTATION IN PATIENTS RECEIVING PEMBROLIZUMAB PLUS AXITINIB FOR METASTATIC RENAL CELL CARCINOMA: PRELIMINARY RESULTS FROM A RANDOMIZED, DOUBLE‐BLIND, PLACEBO‐CONTROLLED TRIAL (TACITO TRIAL)

S. Porcari^1,2^, C. Ciccarese^1,2^, G. Piccinno^3^, D. Rondinella^1,2^, A. Severino^1,2^, S. Buti^4^, G. Fornarini^5^, F. Primi^6^, L. Stumbo^7^, D. Arduini^1,2^, F. Asnicar^8^, F. Pinto^8^, F. Armanini^8^, G. Quaranta^1,2^, I. Venturini^1,2^, L. Masucci^1,2^, M. Sanguinetti^1,2^, A. Gasbarrini^1,2^, G. Tortora^1,2^, G. Cammarota^1,2^, N. Segata^8^, R. Iacovelli^1,2^, G. Ianiro ^1,2^

^1^ *Catholic University of Rome, Rome, Italy*, ^2^ *Fondazione Policlinico Universitario “A. Gemelli” IRCCS, Rome, Italy*, ^3^ *University of Trento, Trento, Italy*, ^4^ *University of Parma, Parma, Italy*, ^5^ *Genova University, Genova, Italy*, ^6^ *Belcolle Hospital, Viterbo, Italy*, ^7^ *Campus Biomedico University, Rome, Italy*, ^8^ *Centre for Integrative Biology, University of Trento, Trento, Italy*

**Contact E‐Mail Address**: gianluca.ianiro@hotmail.it

**Introduction**: Immune checkpoint inhibitors (ICIs), alone or in combination with tyrosine‐kinase inhibitors (TKIs), have changed the therapeutic landscape of several cancers but response is still unsatisfactory in a considerable (>30%) rate of patients. Increasing evidence shows that gut microbiome composition associates with response to ICIs in patients with epithelial cancers. Preliminary evaluations also suggest that fecal microbiota transplantation (FMT) may enhance response to ICIs in patients with melanoma, but randomized studies to confirm these results as well as findings from other epithelial cancers, are still unavailable.

**Aims & Methods**: Our aim is to investigate by a randomized, double‐blind, placebo‐controlled trial (TACITO trial—NCT04758507) whether targeted FMT (from donors who respond to ICIs) is effective in improving response to combined ICIs/TKIs first‐line therapy in patients with advanced renal cell carcinoma (aRCC).

Fifty patients with aRCC, who were starting or have started no earlier than 8 weeks treatment with axitinib + pembrolizumab as first‐line therapy were randomized 1:1 to receive donor FMT (d‐FMT) or placebo FMT (p‐FMT) for three times in 6 months. Both groups underwent the first procedure with a colonoscopy followed by capsulized FMT after 90 and 180 days, respectively. Patients in the d‐FMT arm received feces from a patient with mRCC who had achieved a complete and long‐lasting response to ICIs selected by following protocols recommended by international guidelines.

Patients were followed up 7, 15, 30, 90, 180, 270, and 360 days after randomization for clinical evaluation and collection of biological samples. The primary endpoint was the progression‐free survival (PFS) at 12 months. Secondary endpoints were as follows: median PFS, overall survival (OS), objective response rate (ORR), adverse events after FMT, and microbiome changes after FMT.

Sample size calculation was based on the hypothesis that FMT can provide a 20% improvement of the 12‐month PFS rate when associated to standard of care (SOC)—from 60%, that is the reported 12‐month PFS for SOC‐ to 80%.

**Results**: Fifty patients were randomized to d‐FMT (International mRCC Database Consortium [IMDC] risk: 28% favorable, 72% intermediate–poor) or p‐FMT (32% favorable, 68% intermediate–poor). At August 2024, 44 of them completed the follow‐up and were evaluated for the primary endpoint (24 in the d‐FMT arm and 20 in the p‐FMT arm). The 12‐month PFS rate was 66.7% in the d‐FMT arm versus 35.0% in the p‐FMT arm (*p* = 0.036). In the overall population, after a median follow‐up of 28.0 months, the median PFS was 14.2 months (95% CI, 0.9–27.6) in the d‐FMT arm versus 9.2 months (95% CI, 3.0–15.4) in the p‐FMT arm, and the median OS were not reached versus 25.3 (95%CI, 17.1–33.6) months with d‐FMT and p‐FMT, respectively. The ORR was 54% in the d‐FMT arm versus 28% in the p‐FMT arm. Stable disease (SD) was found in 38% of the d‐FMT arm versus 44% of the p‐FMT arm. Disease progression (PD) was observed in 8% of the d‐FMT arm versus 28% of the p‐FMT arm. Only one patient in the p‐FMT arm reported a procedure‐related adverse event (grade 3 oral mucositis) and discontinued capsules assumption.

**Conclusion**: The preliminary results of this unprecedented randomized controlled trial suggest that responder‐derived FMT may improve the efficacy of ICIs + TKIs in patients with aRCC.

**Disclosure**: Nothing to disclose.

## LB02 | FECAL MICROBIOTA TRANSPLANT VERSUS VANCOMYCIN FOR PRIMARY CLOSTRIDIODES DIFFICILE INFECTION—A RANDOMIZED NON‐INFERIORITY TRIAL

F. E. Juul ^1^, M. Bretthauer^1^, P. Holger Johnsen^2^, K. Tonby^3^, J.‐E. Berdal^4^, D. A. L. Hoff^5^, E. Ofstad^6^, A. Abraham^7^, B. Seip^7^, H. Wiig^8^, Ø. Bakken Rognstad^9^, I. Frivold Glad^10^, J. Valeur^11^, A. Nissen‐Lie^12^, E. Ness‐Jensen^13^, K. Aarberg‐Lund^14^, L. Skjevling^2^, K. Hanevik^15^, H. Skudal^16^, E. Melsom^17^, R. Boyar Cetinkaya^18^, T. Cooper^19^, T. Ranheim^20^, E. Middelfart‐Riise^21^, R. Goll^22^, H.‐O. Adami^23^, M. Kalager^24^, M. Løberg^24^, K. Garborg^1^

^1^ *Oslo University Hospital, Rikshospitalet, Oslo, Norway*, ^2^ *University Hospital North Norway, Harstad, Harstad, Norway*, ^3^ *Ringerike Hospital, Hønefoss, Norway*, ^4^ *Akershus University Hospital, Lørenskog, Norway*, ^5^ *Division of Gastorenterology and Hepatology, Department of Medicine, Møre & Romsdal Hospital Trust, Aalesund Hospital, Aalesund, Norway*, ^6^ *Helgelandssykehuset, Bodø, Bodø, Norway*, ^7^ *Gastroenterology, Vestfold Hospital, Tønsberg, Norway*, ^8^ *Department of Gastroenterology, Sørlandet Hospital, Kristiansand, Norway*, ^9^ *Lillehammer Hospital, Lillehammer, Norway*, ^10^ *Lovisenberg Diaconal Hospital, Oslo, Norway*, ^11^ *Lovisenberg Diaconal Hospital, Unger‐Vetlesens institute, Oslo, Norway*, ^12^ *Bærum Hospital, Oslo, Norway*, ^13^ *Department of Public Health and Nursing, HUNT Research Centre, NTNU, Norwegian University of Science and Technology, Levanger, Norway*, ^14^ *Kalnes Hospital, Sarpsborg, Norway*, ^15^ *Haukeland University Hospital, Bergen, Norway*, ^16^ *Telemark Hospital, Skien, Norway*, ^17^ *Department of Medicine, Kristiansund Hospital, Kristiansund, Norway*, ^18^ *Medicine, Diakonhjemmet Hospital, Oslo, Norway*, ^19^ *Stavanger University Hospital, Stavanger, Norway*, ^20^ *Fürst Laboratories, Oslo, Norway*, ^21^ *Molde Hospital, Molde, Norway*, ^22^ *University Hospital North Norway, Tromsø, Tromsø, Norway*, ^23^ *Karolinska Institutet, Stockholm, Sweden*, ^24^ *University of Oslo, Oslo, Norway*

**Contact E‐Mail Address**: f.e.juul@medisin.uio.no

**Introduction**: Antibiotic‐associated colitis due to *Clostridioides difficile* infection is an increasing health problem worldwide. The currently recommended therapy for primary infection is antibiotic treatment with either vancomycin or fidaxomicin. Fecal microbiota transplantation is recommended for recurrent *C. difficile* infection but its benefits in primary *C. difficile* infection are unclear. Our objectives were to investigate if fecal microbiota transplantation is noninferior to standard antibiotic treatment to achieve lasting clinical cure of primary *C. difficile* infection, and to investigate adverse effects related to fecal microbiota transplantation in primary *C. difficile* infection.

**Aims & Methods**: We performed a noninferiority multicenter randomized trial in adult patients in Norway with acute *C. difficile* infection (≥3 loose stools per day and positive *C. difficile* toxin stool test) and no previous *C. difficile* infection. Patients were randomized to either one rectal fecal microbiota transplantation or 10 days oral vancomycin 125 mg four times a day. Each fecal microbiota transplant was made from 50 g feces from healthy donors aged 16–30 at University Hospital of North Norway (Harstad, Norway). Before administration, patients assigned to fecal microbiota transplantation were asked to empty their bladder and rectum. There were no other preparatory procedures nor any sedation for the procedure. The primary endpoint was the proportion of patients with clinical cure (firm stool consistency or ≤3 bowel movements per day) at 14 days and no recurrence within 60 days in modified intention‐to‐treat analyses. The trial was registered at clinicaltrials.gov (ID: NCT03796650).

**Results**: Out of 104 randomized patients, 100 received fecal microbiota transplantation or the first dose of vancomycin and were eligible for analyses. Clinical cure and no disease recurrence was observed in 34 of 51 patients (66.7%; 95% CI: 52.6%–78.3%) with fecal microbiota transplantation versus 31 of 49 (63.3%; 95% CI: 48.9%–75.6%) with vancomycin (difference in treatment success 3.4%; 95% CI: −21.2% to 28.0%; *p*‐value for non‐inferiority of fecal microbiota transplantation vs. vancomycin: 0.001). Clinical cure at day 14 and no recurrence with or without additional treatment was 40 out of 51 (78.4%; 95% CI: 64.9%–87.7%) with fecal microbiota transplantation and 31 out of 49 (63.3%; 95% CI: 48.9%–75.6%) with vancomycin (difference in treatment success 15.2%; 95% CI: −8.0 to 38.3; *p*‐value for non‐inferiority of fecal microbiota transplantation vs. vancomycin: <0.001). There were no differences in adverse events between the groups.

**Conclusion**: Our study shows that fecal microbiota transplantation is safe and noninferior to Vancomycin in primary *C. difficile* infection.

**Disclosure**: F.E. Juul has talked about fecal microbiota transplantation, pro bono, at a scientific meeting organized by Tillotts Pharma. The other authors have no conflicts of interest to declare.

## LB03 | LACKING NEUTRALIZATION AGAINST THE SARS‐COV‐2 JN.1 VARIANT FOLLOWING ORIGINAL MRNA VACCINES IS BOOSTED BY XBB.1.5‐ADAPTED MRNA VACCINES BUT NOT BY INFECTIONS WITH PREVIOUS OMICRON VARIANTS IN PATIENTS WITH INFLAMMATORY BOWEL DISEASE (STAR SIGN STUDY): A PROSPECTIVE MULTICENTER COHORT STUDY

S. Woelfel ^1,2^, J. Dütschler^1,3^, D. Junker^4^, M. König^1^, N. Graf^5^, C. Krieger^1^, S. Truniger^1,3^, V. Oikonomou^6^, G. Leinenkugel^7^, S. Koller^1^, K. Metzger‐Peter^1^, J. Wyss^6^, N. Krupka^6^, N. Frei^1^, W. C. Albrich^8^, M. Friedrich^9^, J. H. Niess^7,10^, N. Schneiderhan‐Marra^4^, A. Dulovic^4^, B. Misselwitz^6,11^, W. Korte^12^, J. J. Bürgi^12^, S. Brand^1^, STAR SIGN Study Investigators

^1^ *Department of Gastroenterology and Hepatology, Cantonal Hospital St. Gallen, St. Gallen, Switzerland*, ^2^ *Max von Pettenkofer Institute of Hygiene and Medical Microbiology, Ludwig Maximilian University of Munich, Faculty of Medicine, Munich, Germany*, ^3^ *Outpatient Clinic, Ambulatory Services Rorschach, Rorschach, Switzerland*, ^4^ *NMI Natural and Medical Sciences Institute at the University of Tübingen, Reutlingen, Germany*, ^5^ *Clinical Trials Unit, Cantonal Hospital St. Gallen, St. Gallen, Switzerland*, ^6^ *Department of Visceral Surgery and Medicine, Inselspital Bern University Hospital, Bern, Switzerland*, ^7^ *Clarunis, Department of Gastroenterology and Hepatology, University Digestive Healthcare Center, Basel, Switzerland*, ^8^ *Division of Infectious Diseases, Infection Prevention & Travel Medicine, Cantonal Hospital St. Gallen, St. Gallen, Switzerland*, ^9^ *Translational Gastroenterology and Liver Unit, University of Oxford, Nuffield Department of Medicine, Oxford, UK*, ^10^ *Department of Biomedicine, Gastroenterology Group University of Basel, Basel, Switzerland*, ^11^ *Medical Clinic II, Ludwig Maximilian University of Munich, Munich, Germany*, ^12^ *Center for Laboratory Medicine, St. Gallen, Switzerland*

**Contact E‐Mail Address**: woelfel@mvp.lmu.de

**Introduction**: The widespread use of COVID‐19 mRNA vaccines marked a turning point during the 2020 coronavirus pandemic and saved millions of lives ever since.^1^ Until today, vaccines continue to protect from severe illness and long‐term ailments associated with COVID‐19 and, therefore, are crucial in controlling the burden of SARS‐CoV‐2 on healthcare systems and personal life quality.^2,3^ Anti‐TNF‐treated patients with inflammatory bowel disease (IBD) are at increased risk for SARS‐CoV‐2 infection due to impaired vaccine immunogenicity.^4–6^ To maintain high levels of immunoprotection, adapted COVID‐19 vaccines are recommended for patients with IBD. However, many IBD patients rely on preexisting immunity elicited by original vaccines or prior infections. We assessed if such immunity sufficiently combats highly immune‐evasive SARS‐CoV‐2 variants causing the current summer 2024 COVID‐19 wave.

**Aims & Methods**: Utilizing two longitudinal cohorts, we evaluated immunity against the SARS‐CoV‐2 variant JN.1 induced by original vaccines (IBD: *n* = 98; healthy: *n* = 48), omicron breakthrough infection (IBD: *n* = 55; healthy: *n* = 57), or XBB.1.5‐adapted vaccines (IBD: *n* = 18). Neutralization and anti‐receptor binding domain (RBD) IgG levels against wild‐type SARS‐CoV‐2 and JN.1 were assessed using multiplex immunoassays. The primary outcome was wild‐type and JN.1 neutralization following three doses of original mRNA vaccines, stratified by immunosuppressive therapy. Secondary outcomes were wild‐type and JN.1 neutralization following third‐dose breakthrough infection or a fourth dose of XBB.1.5‐adapted mRNA vaccines.

**Results**: Following original vaccines, JN.1 neutralization was lower than wild‐type neutralization in all study groups (healthy, anti‐TNF, non‐anti‐TNF; each *p* < 0.001), and most individuals lacked JN.1 neutralization (healthy: 97.9%; anti‐TNF: 98.3%; non‐anti‐TNF: 92.3%). Confounder‐adjusted multivariable modeling strongly associated anti‐TNF therapy with low levels of anti‐JN.1‐RBD IgG (fold‐change 0.48 [95% CI 0.39–0.59]; Table 1). JN.1 neutralization was similar in patients with versus without breakthrough infection (anti‐TNF, non‐anti‐TNF; each *p* > 0.05), and neutralization failure was 100% despite breakthrough infection. XBB.1.5‐adapted vaccines enhanced JN.1 neutralization (*p* < 0.001) and reduced neutralization failure rates in IBD patients (94.4% pre‐vaccination vs. 44.4% post‐vaccination; *p* = 0.003).

**TABLE 1:** Multivariable model with logarithmized levels of anti‐RBD IgG against the JN.1 variant after three original vaccine doses as outcome in patients with IBD and healthy controls. Exponentiated fold‐changes and bootstrapped 95% CIs were exponentiated to show fold‐changes of antibody concentration. Data are based on serum samples collected two to 16 weeks after vaccination.

Variable	*n*/*N*	Fold‐change	95% confidence interval
Anti‐TNF therapy	58/143	0.48	0.39–0.59
Non‐anti‐TNF therapy	38/143	0.95	0.76–1.21
Previous SARS‐CoV‐2 infection	17/143	0.87	0.65–1.20
Time between third‐dose vaccination and sampling (per month)	143/143	0.78	0.67–0.89
Age (per decade)	143/143	0.97	0.90–1.04
Active smoking	26/143	0.80	0.63–0.97
Time between second‐ and third‐dose vaccination (per month)	143/143	1.00	0.93–1.08
Vaccination with mRNA‐1273 (vs. BNT162b2)	27/143	1.18	0.87–1.49

**Conclusion**: Only variant‐adapted vaccines protect against emerging SARS‐CoV‐2 variants. IBD patients and healthy individuals without recent vaccination lack protection against JN.1 and its subvariant KP.3.1.1, which contributes to current COVID‐19 surges. Vaccination campaigns must be intensified to guarantee sufficient immunoprotection in patients with IBD and healthy individuals.

**References**

1. Watson OJ, Barnsley G, Toor J, Hogan AB, Winskill P, Ghani AC. Global impact of the first year of COVID‐19 vaccination: a mathematical modelling study. *Lancet Infect Dis*. 2022;22:1293–1302.
2. Lin DY, Du Y, Xu Y, Paritala S, Donahue M, Maloney P. Durability of XBB.1.5 Vaccines against omicron subvariants. *N Engl J Med*. 2024. 10.1056/NEJMc2402779
3. Lundberg‐Morris L, Leach S, Xu Y, Martikainen J, Santosa A, Gisslén M, et al. Covid‐19 vaccine effectiveness against post‐covid‐19 condition among 589722 individuals in Sweden: population based cohort study. *BMJ*. 2023. 10.1136/bmj‐2023‐076990
4. Liu Z, Le K, Zhou X, Alexander JL, Lin S, Bewshea C, et al. Neutralising antibody potency against SARS‐CoV‐2 wild‐type and omicron BA.1 and BA.4/5 variants in patients with inflammatory bowel disease treated with infliximab and vedolizumab after three doses of COVID‐19 vaccine (CLARITY IBD): an analysis of a prospective multicentre cohort study. *Lancet Gastroenterol Hepatol*. 2023;8:145–156.
5. Woelfel S, Dütschler J, König M, Graf N, Oikonomou V, Krieger C, et al. Systemic and T cell‐associated responses to SARS‐CoV‐2 immunisation in gut inflammation (STAR SIGN study): effects of biologics on vaccination efficacy of the third dose of mRNA vaccines against SARS‐CoV‐2. *Aliment Pharmacol Ther*. 2023;57:103–116.
6. Woelfel S, Dütschler J, König M, Dulovic A, Graf N, Junker D, et al. STAR SIGN study: Evaluation of COVID‐19 vaccine efficacy against the SARS‐CoV‐2 variants BQ.1.1 and XBB.1.5 in patients with inflammatory bowel disease. *Aliment Pharmacol Ther*. 2023;58:678–691.

**Disclosure**: SB received speaker’s honoraria from Abbvie, Falk Foundation, Ferring, Janssen, Lilly, MSD, Takeda, UCB, and Vifor and participated in advisory boards of Abbvie, BMS, Celgene, Janssen, Lilly, MSD, Pfizer, Pierre Fabre, Roche, Takeda, and UCB. SB has received an educational grant from Takeda. The funders had no role in the design of the study, in the collection, analyses, or interpretation of data, in the writing of the manuscript, or in the decision to publish the results. WCA received funding from the Swiss National Science Foundation (33IC30_201300), Cantonal Hospital St. Gallen, OM Pharma, FUNGINOS, Gilead, received payment for lectures and presentations by Pfizer, GSK, MSD, Gilead, paid to his institution, received payment for travel to meetings from Pfizer, GSK, Gilead, paid to his institution, and participated in the advisory boards of MSD, Sanofi, Pfizer, GSK, OM Pharma, Moderna, Aurovir Pharma, and Janssen. ST participated in the advisory boards of Takeda, Janssen, BMS, and Medinova. BM has received speaking or traveling fees and/or served on an advisory board with BMS, Abbvie, Takeda, Falk, iQONE, Amgen, MSD, Jansen, Gilead, and has received unrestricted research grants from MSD, BMS and Nestle.

## LB04 | THE VIRAL CODE: INVESTIGATING CROHN'S DISEASE THROUGH THE LENS OF MOLECULAR MIMICRY, CLONAL EXPANSION AND NEAR‐SEQUENCE SIMILARITY

E. Beltazar ^1^, T. Patel^1^, O. Siddique^1^, E. Beltazar^1^, F. Rouf^2^, R. Alam^1^, P. Tagdiwala^1^, H. Panchal^1^, P. Sivakumar^1^, J. Mantilla^1^, N. Sirajudeen^1^

^1^ *Medicine, University College London, London, UK*, ^2^ *Medicine, University of East Anglia Norwich Medical School, Norwich, UK*

**Contact E‐Mail Address**: emmanuelbeltazar1402@gmail.com

**Introduction**: The gut virome has been previously linked to the development of Crohn's disease (CD), a type of Inflammatory Bowel Disease.^1^ Recent findings have implicated the expansion of *Caudovirales* as a potential trigger for CD.^2^ However, despite speculations regarding molecular mimicry, there has been insufficient investigation into the causal connection between Caudovirales and CD. This lack of understanding stems from the absence of robust data on the immunological effects of Caudovirales and the technical difficulties associated with analyzing protein structures, which have impeded efforts to establish a definitive causal relationship. Consequently, there remains a critical gap in our comprehension of how the virome contributes to CD.

**Aims & Methods**: In this study, we aimed to investigate the differences in T‐cell dynamics between patients with Crohn's disease (CD) and normal controls to explore the potential mechanistic link between CD and Caudovirales. We considered the following three possible mechanisms: oligoclonal expansion, typically associated with antigen‐driven clonal expansion; polyclonal expansion, which can give rise to self‐antigen targeting cells; and the absence of T‐cell expansion, which would suggest the involvement of other immune mechanisms. By examining T‐cell dynamics, we sought to gain insights into the immune processes at play in the pathophysiology of CD. To investigate clonal expansion, we used pcDelta, a near‐coincidence statistic originally developed for evaluating T‐cell receptor (TCR) clonotype skewing following antigen exposure. The amount of near‐sequence similarity is a surrogate for the degree of clonal expansion to a particular antigen and can thus be used for this purpose.

CDR3 sequences of the beta chain of T‐cell receptors (TCRs) specific to various viral antigens were obtained from the Immune Epitope Database (IEDB), a rigorously curated repository of TCR sequences with annotated antigen specificities. Additionally, disease‐specific CDR3 sequences were compiled from a comprehensive review that consolidated TCR clonotypes isolated from individuals diagnosed with Crohn's disease (CD) drawn from multiple studies. Using pcDelta, we quantified the near‐sequence similarity at the amino acid level between TCRs from CD patients and those specific to various viral antigens, comparing these similarities to those observed in healthy controls. To account for the varying numbers of epitope‐specific TCRs, subsampling was performed, and a pcDelta value was calculated for each sample.

**Results**: Our results showed significantly increased near‐sequence similarity (*p*‐value <0.001) across all viral antigens for the CDR3 sequences of TCR isolated from healthy controls as opposed to patients with CD, suggesting oligoclonal expansion to some unknown antigens in patients with CD. Relative T‐cell expansion between the various viral antigens within each group was preserved, consistent with the above findings.

**Conclusion**: The significant decrease in near‐sequence similarity between the CDR3 sequences of patients with CD and viral antigens as opposed to healthy controls is suggestive of oligoclonal expansion, consistent with the idea in the literature of molecular mimicry. However the source of the antigen leading to oligoclonal expansion is yet to be identified. Isolating T‐cells from individuals exposed to *Caudovirales* and analyzing the TCR Repertoire might prove useful in identifying the source antigen. Future investigations could also employ structural modeling or functional assays to elucidate the impact of molecular mimicry in CD.

**References**

1. Diculescu M, Manuc T‐E, Manuc M. Recent insights into the molecular pathogenesis of Crohn's disease: A review of emerging therapeutic targets. *Clin Exp Gastroenterol*. 2016:59. 10.2147/ceg.s53381
2. Errico C, Massimino L, Facoetti A, Nicolò S, Cagliani S, Spanò S, et al. P057 caudovirales may trigger molecular mimicry mechanisms in Crohn's disease. *J Crohns Colitis*. 2024;18(Supplement_1):i325–i325. 10.1093/ecco‐jcc/jjad212.0187

**Disclosure**: Nothing to disclose.

## LB05 | STATIN USE IS ASSOCIATED WITH A LESS SEVERE DISEASE COURSE IN INFLAMMATORY BOWEL DISEASE: A NATIONWIDE POPULATION‐BASED COHORT STUDY 2006–2020

H. Khalili^1,2^, A. Forss ^3,4^, J. Söderling^3^, G. Bröms^3,5^, C. Eriksson^3,6^, J. Sun^7^, J. F. Ludvigsson^7,8,9^, O. Olén^3,10,11^

^1^ *Harvard Medical School, Boston, Massachusetts, USA*, ^2^ *Division of Gastroenterology, Department of Medicine, Massachusetts General Hospital, Boston, Massachusetts, USA*, ^3^ *Clinical Epidemiology Division, Department of Medicine Solna, Karolinska Institutet, Stockholm, Sweden*, ^4^ *Department of Gastroenterology, Dermatovenereology and Rheumatology, Centre for Digestive Health, Karolinska University Hospital, Stockholm, Sweden*, ^5^ *Division of Gastroenterology, Department of Specialist Medicine, Danderyd Hospital, Stockholm, Sweden*, ^6^ *Department of Gastroenterology, Faculty of Medicine and Health, Örebro University, Örebro, Sweden*, ^7^ *Department of Medical Epidemiology and Biostatistics, Karolinska Institutet, Stockholm, Sweden*, ^8^ *Department of Paediatrics, Örebro University Hospital, Örebro, Sweden*, ^9^ *Division of Digestive and Liver Disease, Department of Medicine, Columbia University Medical Center, New York, New York, USA*, ^10^ *Sachs' Children and Youth Hospital, Stockholm South General Hospital, Stockholm, Sweden*, ^11^ *Department of Clinical Science and Education, Stockholm South General Hospital, Karolinska Institutet, Stockholm, Sweden*

**Contact E‐Mail Address**: anders.forss@ki.se

**Introduction**: Statins have been associated with a reduced risk of incident inflammatory bowel disease (IBD). The effect of statins on disease progression in IBD is largely unknown.

**Aims & Methods**: We aimed to investigate the effect of statins on disease progression in UC and CD. We performed a nationwide register‐based cohort study (2006–2020) including 19,788 adults (≥18 years) with incident ulcerative colitis (UC) and 12,582 with incident Crohn's disease (CD). Of these, 1733 with UC and 962 with CD were identified as incident statin users after UC or CD diagnosis. Index date was set to the date of first dispensed statin prescription of ≥30 cumulative defined daily doses after diagnosis of UC or CD. Individuals with any statin prescription within 12 months prior to and up to 6 months after diagnosis were excluded. After 1:1 propensity score matching (direct matching by IBD subtype, sex, age at diagnosis, calendar year of diagnosis, and level of education, with propensity scores adjusted for comorbidities and co‐medications), we compared statin users with nonusers applying Cox proportional hazards modeling to estimate the risk of IBD‐related surgery, hospitalizations, and disease flares (any of systemic corticosteroid prescription, start of immunomodulator, or start/switch to anti‐tumor necrosis factor treatment). We reported incidence rates and multivariable‐adjusted hazard ratios (aHRs) with 95% confidence intervals (CIs). For outcomes with statistically significant estimates, we calculated the number needed to treat (NNT).

**Results**: At start of follow‐up, the median age for statin users with UC was 61 years (interquartile range [IQR] 52–69) and 59 years (IQR 49–67) for statin users with CD. In UC, the majority were males (54.9%), while in CD the sex distribution was even (males 49.5% vs. females 50.5%). During a median follow‐up of 3.4 years after first statin exposure, we observed a reduced risk of IBD‐related surgery in statin users compared with nonusers with UC (aHR: 0.55 [95% CI: 0.31–0.97]) and CD (0.54 [0.33–0.88]). The NNTs to avoid one IBD‐related surgical event per year of statin treatment were 345 and 161 for UC and CD, respectively. The risks of hospitalizations (0.68 [0.51–0.91]) and disease flares (0.86 [0.77–0.97]) in statin users were reduced for UC but not for CD (0.78 [0.56–1.09] and 1.02 [0.88–1.19]). For UC, NNTs for hospitalizations and disease flares were 145 and 15 per year of statin treatment.

**Table:** TABLE 1 Incidence rates, hazard ratios and number needed to treat of inflammatory bowel disease related surgery, hospitalizations, and disease flares in individuals with ulcerative colitis and Crohn's disease with and without statin treatment 2006–2020.

Outcome	*N*		*N* events		Incidence rate per 1000 person‐years (95% CI)		Hazard ratio* (95% CI)	Adjusted hazard ratio** (95% CI)	NNT*** per 1 year of statin treatment
	Statin users	Nonstatin users	Statin users	Nonstatin users	Statin users	Nonstatin users			
IBD‐related surgery									
UC	1733	1733	24 (1.4%)	34 (2.0%)	3.4 (2.1–4.8)	6.3 (4.2–8.5)	0.56 (0.33–0.95)	0.55 (0.31–0.97)	345
CD	962	962	36 (3.7%)	48 (5.0%)	9.2 (6.2–12.2)	15.4 (11.0–19.7)	0.59 (0.39–0.90)	0.54 (0.33–0.88)	161
IBD‐hospitalization									
UC	1733	1733	113 (6.5%)	121 (7.0%)	17.0 (13.9–20.2)	23.9 (19.6–28.1)	0.77 (0.59–0.99)	0.68 (0.51–0.91)	145
CD	962	962	76 (7.9%)	84 (8.7%)	20.3 (15.8–24.9)	28.2 (22.2–34.2)	0.78 (0.57–1.06)	0.78 (0.56–1.09)	‐
Disease flare									
UC	1733	1733	819 (47.3%)	825 (47.6%)	207.4 (193.2–221.6)	275.0 (256.2–293.8)	0.85 (0.77–0.94)	0.86 (0.77–0.97)	15
CD	962	962	496 (51.6%)	478 (49.7%)	245.5 (223.9–267.1)	292.3 (266.1–318.5)	0.96 (0.85–1.08)	1.02 (0.88–1.19)	

Abbreviation: NNT, number needed to treat.

*Unadjusted. **Conditioned on the matching set and calculated with stratified Cox regression. ***Only calculated for adjusted hazard ratios that reached statistical significance.

**Conclusion**: In this population‐based study, statins were associated with a reduced risk of IBD‐related surgery, hospitalizations, and disease flares in individuals with UC but only with surgery in individuals with CD.

**Disclosure**: Dr Khalili has received consulting fees from Takeda and Aditum Bio and has received grant funding from Takeda and Pfizer. Dr Forss has served as a speaker and advisory board member for Janssen Corporation and Tillotts Pharma. Dr Eriksson received grant support/lecture fee/advisory board from BMS, Takeda, Janssen Cilag, Pfizer, Abbvie. Dr Ludvigsson has coordinated an unrelated study on behalf of the Swedish IBD quality register (SWIBREG). This study received funding from Janssen Corporation. He has also received financial support from MSD to develop a paper reviewing national healthcare registers in China, and has an ongoing collaboration with Takeda on celiac disease. Dr Olén has been PI on projects at Karolinska Institutet financed by grants from Janssen, Pfizer, AbbVie, Takeda, Ferring, and Bristol Myers Squibb. Karolinska Institutet has also received fees for lectures and participation by him on advisory boards from Janssen, Ferring, Galapagos, Bristol Myer Squibb, Takeda, and Pfizer. Dr Olén also reports grants from Pfizer, AbbVie, Galapagos, and Janssen in the context of national safety monitoring programs. Drs Söderling, Sun, Bröms and Ericsson declare no conflicts of interest.

## Crohn's disease hot of the press: Late‐breaking abstracts 10:00–11:00/A1

## LB06 | ETRASIMOD FOR MODERATELY TO SEVERELY ACTIVE CROHN'S DISEASE: RESULTS FROM THE EXTENSION PERIOD OF A PHASE 2 STUDY

G. R. D'Haens ^1^, M. C. Dubinsky^2^, L. Peyrin‐Biroulet^3,4,5,6,7,8^, S. Danese^9^, B. E. Sands^10^, A. Yarur^11^, M. V. Chiorean^12^, I. Modesto^13^, D. Branquinho^13^, A. McDonnell^14^, M. Kudela^15^, L. Villa‐Caballero^16^, G. Gu^16^, H. Tan^17^, C. Su^18^, S. Schreiber^19^, B. G. Feagan^20,21^, S. Vermeire^22^

^1^ *Department of Gastroenterology and Hepatology, Amsterdam University Medical Centers, Amsterdam, Netherlands*, ^2^ *Icahn School of Medicine at Mount Sinai, Susan and Leonard Feinstein IBD Center, New York, New York, USA*, ^3^ *Department of Gastroenterology, Nancy University Hospital, Vandœuvre‐lès‐Nancy, France*, ^4^ *INSERM, NGERE, University of Lorraine, Nancy, France*, ^5^ *INFINY Institute, Nancy University Hospital, Vandœuvre‐lès‐Nancy, France*, ^6^ *FHU‐CURE, Nancy University Hospital, Vandœuvre‐lès‐Nancy, France*, ^7^ *Paris IBD Center, Groupe Hospitalier privé Ambroise Paré ‐ Hartmann, Neuilly sur Seine, France*, ^8^ *Division of Gastroenterology and Hepatology, McGill University Health Centre, Montreal, Quebec, Canada*, ^9^ *Division of Gastroenterology and Endoscopy, IRCCS San Raffaele Hospital and Vita Salute San Raffaele University, Milan, Italy*, ^10^ *Dr. Henry D. Janowitz Division of Gastroenterology, Icahn School of Medicine at Mount Sinai, New York, New York, USA*, ^11^ *Inflammatory Bowel Disease Center and Division of Gastroenterology and Hepatology, Cedars‐Sinai Medical Center, Los Angeles, California, USA*, ^12^ *Swedish Medical Center, Inflammatory Bowel Disease Center, Seattle, Washington, USA*, ^13^ *Pfizer Inc, New York, New York, USA*, ^14^ *Pfizer Ltd, Sandwich, UK*, ^15^ *Pfizer Inc, Cambridge, Massachusetts, USA*, ^16^ *Pfizer Inc, La Jolla, California, USA*, ^17^ *Pfizer Inc, Groton, USA*, ^18^ *Pfizer Inc, Collegeville, Pennsylvania, USA*, ^19^ *Department of Internal Medicine I, University Hospital Schleswig‐Holstein, Kiel University, Kiel, Germany*, ^20^ *Division of Gastroenterology, Department of Medicine, Western University, London, Ontario, Canada*, ^21^ *Alimentiv Inc, London, Ontario, Canada*, ^22^ *Department of Gastroenterology and Hepatology, University Hospitals Leuven, Leuven, Belgium*

**Contact E‐Mail Address**: g.dhaens@amsterdamumc.nl

**Introduction**: Etrasimod is an oral, once‐daily, and selective sphingosine 1‐phosphate_1,4,5_ receptor modulator. Within the phase 2/3 CULTIVATE trial (NCT04173273), we report extension data on the efficacy, safety, and tolerability of etrasimod in patients with moderately to severely active Crohn's disease (CD) from substudy A, a phase 2, randomized, and double‐blind study.

**Aims & Methods**: Adults (18–80 years) who completed the 14‐week induction phase (data reported previously^1^) were eligible for the 52‐week extension period. Patients receiving etrasimod 3 mg during induction continued this treatment within the extension period, regardless of response. Etrasimod 2 mg responders at week 14 continued treatment during the extension period while nonresponders were re‐randomized (1:1) to etrasimod 2 or 3 mg. Two analyses were performed based on two approaches for patients entering extension: treat‐through (TT; outcomes reported for all randomized patients regardless of week 14 response) and responders only (RO; outcomes reported for week 14 responders only). Both used nonresponder imputation. The primary endpoint was the achievement of endoscopic response (defined by endoscopic remission or ≥50% decrease in Simple Endoscopic Score in Crohn's Disease) at week 52; other endpoints included the achievement of clinical remission based on Crohn's Disease Activity Index (CDAI; <150) and based on PRO2 (<8) at week 52 (Table). Treatment‐emergent adverse events (TEAEs) were reported for the safety set (all randomized patients who received ≥1 dose).

**Results**: In total, 65 patients (safety set) were enrolled in the extension period with 38 completing the study; patient demographics and baseline characteristics are shown in the Table. For the TT analysis, 14.3% and 19.5% of patients receiving 2 mg or 3 mg etrasimod, respectively, achieved endoscopic response, 28.6% and 34.1% achieved CDAI remission, and 19.0% and 36.6% achieved PRO2 remission at week 52 (Table). Among Week 14 responders (RO), 23.1% and 33.3% of patients receiving 2 or 3 mg etrasimod, respectively, achieved endoscopic response, 46.2% and 54.2% achieved CDAI remission, and 30.8% and 58.3% achieved PRO2 remission at week 52 (Table). Most TEAEs were mild or moderate. Nine TEAEs led to discontinuation; one was deemed treatment‐related (Table). No deaths or TEAEs of macular edema or malignancies were reported.

**Table:** TABLE Baseline demographics and clinical characteristics of patients entering the extension period, proportions of patients achieving efficacy endpoints at week 52 and summary of safety data.

	Etrasimod 2 mg QD	Etrasimod 3 mg QD
	(*N* = 28)	(*N* = 37)
Demographics and baseline clinical characteristics (extension period)		
Age, mean (SD), years	41.8 (12.8)	41.6 (14.2)
Sex, female (%)	53.6	43.2
Extent of disease (%)		
Ileal disease	17.9	24.3
Colonic disease	39.3	21.6
Ileocolonic disease	42.9	54.1
Baseline CDAI, mean (SD)	338.6 (81.2)	315.7 (61.5)
Baseline SES‐CD, mean (SD)	12.3 (7.3)	10.7 (5.9)
Baseline systemic corticosteroids (%)	39.3	35.1
Prior use of thiopurines/methotrexate^a^ (%)	46.4	56.8
Number of prior biologics^a^ (%)		
0	50.0	40.5
1	21.4	18.9
2	10.7	13.5
≥3	17.9	27.0
Efficacy endpoints at week 52		
Endoscopic response^b^ [*n*/*N* (%)]		
Treat‐through^c^	14.3 (6/42)	19.5 (8/41)
Responders only^d^	23.1 (6/26)	33.3 (8/24)
Clinical remission CDAI^e^ [*n*/*N* (%)]		
Treat‐through^c^	28.6 (12/42)	34.1 (14/41)
Responders only^d^	46.2 (12/26)	54.2 (13/24)
Clinical remission PRO2^f^ [*n*/*N* (%)]		
Treat‐through^c^	19.0 (8/42)	36.6 (15/41)
Responders only^d^	30.8 (8/26)	58.3 (14/24)
Summary of safety (extension period)		
Any TEAE [*n* (%)]	22 (78.6)	33 (89.2)
Any serious TEAE [*n* (%)]	4 (14.3)	7 (18.9)
TEAEs leading to study treatment discontinuation [*n* (%)]	3 (10.7)	6 (16.2)^g^
TEAEs leading to death [*n* (%)]	0	0
Most frequently reported TEAEs^h^ [*n* (%)]		
CD (worsening of/flare)	7 (25.0)	8 (21.6)
Headache	3 (10.7)	6 (16.2)
Arthralgia	3 (10.7)	5 (13.5)
COVID‐19	3 (10.7)	9 (24.3)
TEAEs of special interest^i^ [*n* (%)]		
Severe infections (CTCAE ≥3) [*n* (%)]	1 (3.6)^j^	2 (5.4)^k^
Opportunistic infections [*n* (%)]		
Ophthalmic herpes zoster^l^	0	1 (2.7)
Macular edema [*n* (%)]	0	0
Cardiovascular events [*n* (%)]		
1st degree AV block	0	2 (5.4)
2nd degree AV block, Mobitz type I^m^	0	0
Bradycardia^n^	1 (3.6)	0
Hypertension	0	1 (2.7)

Abbreviations: AV, atrioventricular; CD, Crohn's disease; CDAI, Crohn's Disease Activity Index; COVID‐19, coronavirus disease 2019; CTCAE, Common Terminology Criteria For Adverse Events; *N*, number of patients in group; *n*, number of patients; PRO, patient‐reported outcome; PRO2, patient‐reported outcome 2; QD, once daily; SD, standard deviation; SES‐CD, Simple Endoscopic Score in Crohn's Disease; and TEAE, treatment‐emergent adverse event.

^a^ Thiopurines/methotrexate and biologics were discontinued prior to randomisation.

^b^ Endoscopic response defined as endoscopic remission (SES‐CD ≤4 and ≥2‐point reduction from baseline with no subscore >1) or ≥50% decrease in SES‐CD.

^c^ All randomized patients at baseline who received ≥1 dose of etrasimod (*N* = 42 in the etrasimod 2 mg group; *N* = 41 in the etrasimod 3 mg group).

^d^ All patients who were responders at Week 14 (*N* = 26 in the etrasimod 2 mg group; *N* = 24 in the etrasimod 3 mg group).

^e^ CDAI remission defined as CDAI <150.

^f^ PRO2 remission defined as a PRO score <8.

^g^ One patient discontinued due to TEAE which was deemed by the investigator as related to etrasimod 3 mg. All other TEAEs that led to discontinuation were related to CD.

^h^ Occurred in >10% of patients in both etrasimod 2 and 3 mg groups.

^i^ Defined as a subset of TEAEs that met the criteria of the sponsor's medical review, and include cardiovascular events, macular edema, and infections.

^j^ One event of severe COVID‐19.

^k^ One event of severe COVID‐19 and one event of severe anal abscess.

^l^ Event had onset 10 days after the patient had discontinued etrasimod treatment and had started on monoclonal antibody treatment combined with prednisone for worsening CD. The event was assessed as unrelated to study treatment by the investigator.

^m^ No TEAEs of 2nd degree AV block, Mobitz type II or higher were reported.

^n^ Event was non‐serious (Grade 1) and did not require intervention.

^o^Responders were required to achieve clinical response CDAI (clinical remission CDAI or ≥100‐point decrease from baseline in CDAI) or endoscopic response.

**Conclusion**: Both etrasimod doses remained well tolerated with no new safety signals. This non‐placebo‐controlled study suggests that etrasimod may be effective in moderately to severely active CD, with higher efficacy at the 3 mg dose. Placebo‐controlled studies are ongoing.

**Reference**

1. D'Haens G, Dubinsky MC, Peyrin‐Biroulet L, Danese S, Sands BE, Wolf DC, et al. P632 Etrasimod induction therapy in moderately to severely active Crohn's disease: Results from a phase 2, randomised, double‐blind substudy. *J Crohns Colitis*. 2023;17:i764–i765.

**Disclosure**: GD, Advisor: AbbVie, Alimentiv, AstraZeneca, BI, BMS, Celltrion, Cosmo, Lilly, Galapagos, GSK, J&J, Pfizer, Polpharma, Prometheus, Takeda, Tillotts, and Ventyx. Speaker fees: AbbVie, BI, Celltrion, Lilly, J&J, Pfizer, Takeda, and Tillotts; MCD, Consulting fees: AbbVie, Abivax, Arena, AstraZeneca, BMS, Lilly, Galapagos, Genentech, Gilead, Janssen, Merck, Pfizer, Prometheus, Prometheus Laboratories, Sphyre, Takeda, and UCB. Shareholder/Royalties: Trellus Health. Directorship/Ownership interest: Trellus Health. LPB, Consulting fees: AbbVie, Abivax, Alimentiv, Alma Bio, Amgen, Applied Molecular Transport, Arena, Biogen, BMS, Celltrion, CONNECT Biopharm, Cytoki, Enthera, Ferring, Fresenius Kabi, Galapagos, Genentech, Gilead, Gossamer Bio, GSK, HAC, IAG Image Analysis, Index, Inotrem, Janssen, Lilly, Medac, Mopac, Morphic, MSD, Norgine, Novartis, OM, ONO, OSE, Pandion, Par’Immune, Pfizer, Prometheus, Protagonist, Roche, Roivant, Samsung, Sandoz, Sanofi, Takeda, Theravance, Thermo Fisher, Tigenix, Tillotts, Viatris, Vifor, Vectivbio, Ventyx, and Ysopia. Grants: Celltrion, Fresenius Kabi, and Takeda. Lecture/speaker bureau: AbbVie, Amgen, Arena, Biogen, Celltrion, Ferring, Galapagos, Genetech, Gilead, Janssen, Lilly, Medac, MSD, Pfizer, Sandoz, Takeda, Tillotts, Viatris, and Vifor; SD, Lecture/speaker fees: AbbVie, Amgen, Ferring, Gilead, Janssen, Mylan, Pfizer, and Takeda. Consulting/advisory fees: AbbVie, Allergan, Amgen, AstraZeneca, Biogen, BI, Celgene, Celltrion, Ferring, Gilead, Hospira, Janssen, J&J, MSD, Mundi, Pfizer, Roche, Sandoz, Takeda, TiGenix, UCB, and Vifor. Directorship/Ownership: Gastroenterology and Endoscopy. BES, Consulting fees: AbbVie, Abivax, Adiso, Agomab, Alimentiv, Amgen, AnaptysBio, Arena, Artugen, AstraZeneca, Biora, BI, Boston, BMS, Calibr, Celgene, Celltrion, ClostraBio, Enthera, Equillium, Evommune, Ferring, Fresenius Kabi, Fzata, Galapagos, Genentech/Roche, Gilead, GSK, Gossamer Bio, Index, Innovation, Janssen, Kaleido, Kallyope, Lilly, Merck, Mobius Care, Morphic, MRM Health, Nexus, Nimbus Discovery, Odyssey, Palisade Bio, Pfizer, Progenity, Prometheus, Prometheus Laboratories, Protagonist, Q32 Bio, Rasayana, Sun, Surrozen, Takeda, Target RWE, Teva, Theravance, TLL, TR1X, Union, and Ventyx. Speaking fees: AbbVie, Abivax, BMS, Janssen, Lilly, Pfizer, and Takeda. Research grants: BMS, Janssen, Pfizer, and Takeda. Stock and Stock options: Ventyx. Other support: Abivax, BMS, Lilly, Janssen, Pfizer, and Takeda. AY, Consulting fees: AbbVie, Arena, BMS, Pfizer, and Takeda. Lecture fees: AbbVie and BMS. MVC, Grant support: BMS, Janssen, Pfizer, and Novartis. Consulting fees: AbbVie, Arena, BMS, Celltrion, Fresenius Kabi, Janssen, Lilly, Medtronic, Pfizer, Prometheus, and Takeda. Speaker fees: AbbVie, BMS, Janssen, Lilly, Medtronic, Pfizer, and Takeda. IM, DB, AM, MK, LVC, GG, HT, CS, Shareholder and employee: Pfizer; LVC Shareholder and employee: BMS; SS, Lecture/speaker fees: AbbVie, Arena, Biogen, BMS, Celgene, Celltrion, Falk, Fresenius, Janssen, MSD, Pfizer, and Takeda. Consulting/advisory fees: AbbVie, Arena, Biogen, BMS, Celgene, Celltrion, Falk, Fresenius, Gilead, IMAB, Janssen, MSD, Mylan, Pfizer, Protagonist, Provention Bio, Takeda, Theravance, and Ventyx. BF, Senior Scientific Director: Alimentiv. Speaker fees: AbbVie, Janssen, and Takeda. Consulting/advisory board: AbbVie, AbolerIS, AgomAB, Allianthera, Amgen, AnaptysBio, Applied Molecular Transport, Arena, Atomwise, Avoro Capital Advisors, BioJamp, Biora, BI, Boxer, Celgene/BMS, Celsius, Connect, Cytoki, Disc Medicine, Duality, EcoR1, Lilly, Equillium, Ermium, First Wave, First Word Group, Galapagos, Galen Atlantica, Genentech/Roche, Gilead, Gossamer, GSK, Hinge Bio, Hot Spot, Index, Imhotex, Immunic, JAKAcademy, Janssen, Japan Tobacco, Kaleido, Landos, Leadiant, L.E.K. Consulting, Lenczner Slaght, LifeSci Capital, Lument AB, Millennium, MiroBio, Morgan Lewis, Morphic, Mylan, OM, Origo, Orphagen, Pandion, Pendopharm, Pfizer, Play to Know AG, Progenity, Prometheus and Diagnostics, Protagonist, PTM, Q32 Bio, Rebiotix, REDX, Roche, Sandoz, Sanofi, Seres, Silverback, Surrozen, Takeda, Teva, Thelium, Tigenix, Tillotts, Ventyx, VHSquared Ltd, Viatris, Ysios, Ysopia, and Zealand. Shareholder: Gossamer; SV, Lecture/speaker fees: AbbVie, Dr. Falk, Ferring, Galapagos, Hospira, Janssen, MSD, Takeda, and Tillotts. Consulting/advisory fees: AbbVie, AbolerIS, Alimentiv, Arena, AstraZeneca, Avaxia, BMS, BI, Celgene, CVasThera, Dr Falk, Lilly, Ferring, Galapagos, Genentech/Roche, Gilead, Hospira, Imidomics, Janssen, J&J, Materia Prima, MiroBio, Morphic, MrMHealth, MSD, Mundi, Pfizer, Prodigest, Progenity, Prometheus, Robarts Clinical Trials, Second Genome, Shire, Surrozen, Takeda, Theravance, Tillotts AG, and Zealand. Grant/research support: AbbVie, Galapagos, Janssen, MSD, Pfizer, and Takeda.

## LB07 | HIGH ULTRA‐PROCESSED FOODS CONSUMPTION IS ASSOCIATED WITH CLINICAL EXACERBATION IN PATIENTS WITH CROHN'S DISEASE IN REMISSION—A PROSPECTIVE COHORT STUDY

C. Sarbagili Shabat ^1,2^, S. Zelber‐Sagi^1,3^, N. Fliss‐Isakov^1,4^, A. Hirsch^1,2^, Y. Ron^1^, L. S. Grinshpan^3^, N. A. Cohen^1^, H. Leibovitzh^1^, T. Thurm^1,2^, N. Maharshak^1,2^

^1^ *IBD Unit, Department of Gastroenterology and Liver Diseases, Tel‐Aviv Medical Center, Tel Aviv, Israel*, ^2^ *The Faculty of Medical and Health Sciences, Tel‐Aviv University, Tel Aviv, Israel*, ^3^ *School of Public Health, University of Haifa, Haifa, Israel*, ^4^ *Department of Health, School of Public Health, Faculty of Medicine, Tel Aviv University, Tel Aviv, Israel*

**Contact E‐Mail Address**: ibd.chen@gmail.com

**Introduction**: Diet has been suggested to impact the clinical course of Crohn's disease (CD). However, the importance of food processing level is uncertain.

**Aims & Methods**: We aimed to evaluate whether ultra‐processed foods (UPFs) are associated with increased risk of relapse amongst patients with CD.

In this prospective cohort study, adult CD patients in clinical remission (Harvey Bradshaw index, HBI < 5) were followed every 3 months, for 1 year or until clinical relapse (HBI > 4). Dietary intake was assessed using a food frequency questionnaire (FFQ) and using a dedicated validated processed food questionnaire (PFQ) to evaluate UPFs consumption. Multivariable Cox proportional hazards were used to estimate the adjusted Hazard Ratio of disease relapse according to high or low UPFs consumption.

**Results**: A total of 111 CD patients in clinical remission with a mean age of 37.9 ± 14.0 years entered the final analysis. Over a median follow‐up duration of 12 months [interquartile range, 8–12], 24 patients (21.6%) experienced a clinical relapse. A higher intake of UPFs was found to be associated with an increased risk of relapse occurrence (HR = 3.86, 95% CI 1.30–11.47). Clinical relapse was associated with high FC levels at the relapse visit compared to baseline (193.0 μg/g [114.0–807.0], 79.0 μg/g [17.0–274.5], respectively, *p* = 0.002, *n* = 16 for paired analysis). Among different UPF subgroups, ultra‐processed breads, pastries and starches, as well as processed oils and spreads showed the strongest positive associations with CD relapse (HR = 3.27, 95% CI 1.26–8.45; HR = 2.76, 95% CI 1.02–7.45, respectively).

**Table:** TABLE 1 Multivariable analysis^ƚ^ of the association between ultra‐processed food consumption and disease relapse in patients with Crohn's disease.

Ultra‐processed food consumption	Low intake	High intake	HR (95% CI)	*p*‐value
Entire cohort (*N* = 111)				
*N* cases/*N* total	7/57	17/54	3.86 (1.30–11.47)	0.015
No elimination diet sub‐sample (*N* = 103)*				
*N* cases/*N* total	6/51	17/52	4.38 (1.41–13.63)	0.011

The cut‐off for low and high ultra‐processed food consumption was determined by the following median number: 3.6 servings/day.

Abbreviations: CD, Crohn's disease; CI, confidence interval; HR, hazard ratio.

*Patients during elimination diet treatment were excluded from the analysis (*N* = 8).

^ƚ^ Cox model with time‐dependent covariates. HRs are adjusted for: sex, age (years), smoking status, energy intake (kcal/day), saturated fat (% of total kcal), and monounsaturated fatty acids (% of total kcal).

**Conclusion**: Higher consumption of UPFs was associated with an increased risk of relapse in patients with CD in remission. This study, along with future prospective research, can contribute to establish nutritional guidelines aimed at reducing UPFs consumption for patients in clinical remission.

**Disclosure**: Nothing to disclose.

## LB08 | MULTI‐OMICS ANALYSIS OF PERIANAL FISTULIZING CROHN'S DISEASE REVEAL NOVEL THERAPEUTIC TARGETS

S. Cao ^1^, P. Deepak^1^, WU Perianal Crohn's Disease Study Group

^1^ *Gastroenterology, Washington University in St. Louis, St. Louis, Missouri, USA*

**Contact E‐Mail Address**: caos@wustl.edu

**Introduction**: Perianal fistulas occur in ∼30%–40% of patients with Crohn's disease (CD) associated with high morbidity and an impaired quality of life. The management of perianal fistulizing CD (PCD) is a major clinical challenge where its underlying etiology remains elusive. The development of disease‐specific treatment for PCD requires better understanding of its pathogenesis.

**Aims & Methods**: To define the single‐cell landscape of PCD, we recruited patients with (1) PCD (*n* = 25); (2) CD without perianal involvement (NPCD; *n* = 10); and (3) idiopathic perianal fistulas (IPF, or cryptoglandular perianal fistula; *n* = 28). Biopsies were taken from the fistula tracts, openings of the fistulas, and rectal mucosa during examination under anesthesia (EUA) or colonoscopy. Mucosal immune cells were analyzed using mass cytometry (or CyTOF). All cells from PCD and IPF fistula tracts (*n* = 11 or 8) were characterized using single‐cell RNA‐sequencing (scRNA‐seq). Spatial transcriptomics, immunohistochemistry (IHC), and ex vivo cell stimulation were performed to validate the findings. Integrated analysis of published scRNA‐seq datasets was performed to define rectal and ileal cell responses in PCD patients versus those without perianal disease.

**Results**: CyTOF revealed a skewed mucosal immune landscape in PCD compared to NPCD and IPF. PCD expanded Th17 cells in the fistula tracts and IL17‐producing CD8 T cells (Tc17) in the rectum. Altered exhaustion markers CD39 and CD127 were present in both CD4 and CD8 T cells from PCD fistula tracts, fistula openings, and rectum. Regulatory B cells, with immunomodulatory function in the gut, were dramatically diminished in PCD compared to IPF. In addition, PCD fistula tracts, fistula openings, and rectum all exhibited substantially higher CD172+TREM1+ macrophages, which are associated with anti‐TNF resistance in luminal CD. ScRNA‐seq unraveled immune and nonimmune cell compartments in PCD and IPF fistula tracts. PCD fistulas showed hyperactivated pathogenic pathways including interferon (IFN)G response, TNF signaling, and IL6‐JAK‐STAT3 in myeloid cells. Similarly, stromal cells from PCD fistulas exhibited higher activities in IFNG and TNF response, TNF signaling, and epithelial–mesenchymal transition (EMT). Further analysis revealed cellular modules associated with anti‐TNF therapy in PCD patients. In addition to fistula tracts, luminal (rectal and ileal) cells from PCD patients also expressed greater levels of IFNG‐responsive and EMT genes compared to those without perianal disease. In addition, we showed that both fistula tracts and ileal mucosa from PCD patients harbored expanded IFNG+ Th17 cells, which expressed elevated inflammatory mediators including IL17A/F, TNF, GZMA, and CCL5. The findings were independently validated using spatial transcriptomics, IHC, and ex vivo stimulation of PCD fistula‐associated cells.

**Conclusion**: Using multiomics analysis of perianal fistula tracts and luminal tissues from extensive patient cohorts, we revealed previously unknown immune, stromal, and epithelial cell landscapes of PCD. In addition to IL17 signaling, T cell exhaustion, pathogenic macrophages, and proinflammatory/remodeling fibroblasts, our data highlight the pathogenic role of hyperactivated IFNG signaling in both fistula tracts and luminal mucosa of patients with PCD. This study identified IFNG as a potential therapeutic target for PCD.

**Disclosure**: None.

## LB09 | TASTY & HEALTHY FLEXIBLE DIET IS EFFECTIVE FOR INDUCING REMISSION IN CHILDREN AND YOUNG ADULTS WITH MILD–MODERATE CROHN'S DISEASE: A RANDOMIZED CONTROLLED TRIAL WITH EXCLUSIVE ENTERAL NUTRITION AS A COMPARATOR

Y. Aharoni Frutkoff^1^, L. Plotkin^1^, Z. Shavit‐Brunschwig^1^, G. Focht^1^, R. Lev‐Zion^2^, O. Ledder^1^, A. Assa^1^, D. Yogev^1^, E. Orlanski‐Meyer^1^, E. Broide^3^, J. Kierkuś^4^, B. Kang^5^, B. Weiss^6^, M. Aloi^7^, T. Schwerd^8^, A. M. Griffiths^9^, D. Shouval^10^, D. Turner ^1^

^1^ *Shaare Zedek Medical Center, The Juliet Keidan Institute of Paediatric Gastroenterology and Nutrition, Jerusalem, Israel*, ^2^ *Shaare Zedek Medical Center, The Juliet Keidan Institute of Paediatric Gastroenterology and Nutrition, Jerusalem, Israel*, ^3^ *Pediatric Gastroenterology Unit, Assaf Harofeh Medical Center, Tel‐Aviv University, Zerifin, Israel*, ^4^ *Department of Gastroenterology, Hepatology, Feeding Disorders and Pediatrics, The Childrens' Memorial Health Institute, Warsaw, Poland*, ^5^ *Kyungpook National University Children's Hospital, School of Medicine, Kyungpook National University, Daegu, South Korea*, ^6^ *Pediatric Gastroenterology, Edmond and Lily Safra Children's Hospital, The Chaim Sheba Medical Center, Ramat Gan, Israel*, ^7^ *Department of Pediatric Gastroenterology SIGENP IBD Group, Pediatric Gastroenterology and Liver Unit, Sapienza University of Rome, Rome, Italy*, ^8^ *LMU München, Dr von Hauner Children's Hospital, Munich, Germany*, ^9^ *Division of Gastroenterology, Hepatology and Nutrition, The Hospital for Sick Children, University of Toronto, Toronto, Ontario, Canada*, ^10^ *Schneider Children's Medical Center of Israel, Institute of Gastroenterology, Nutrition and Liver Diseases, Petah Tikva, Israel*

**Contact E‐Mail Address**: turnerd@szmc.org.il

**Introduction**: Exclusive enteral nutrition (EEN) is effective for inducing remission in Crohn's disease (CD) but adherence is challenging. CD‐Exclusion Diet (CDED) may be a suitable alternative, but it is structured with mandatory ingredient and requires partial enteral nutrition (PEN). The Tasty&Healthy (T&H) diet excludes processed food, gluten, red meat, and dairy (except for plain yogurt); otherwise, it has no mandatory ingredients and does not require PEN.

**Aims & Methods**: In this clinician‐blinded randomized controlled trial we aimed to assess the tolerability and effectiveness of the T&H diet versus EEN in children and young adults with CD.

Patients aged 6–25 years with mild‐moderate CD were randomized into T&H or EEN for 8‐weeks; both groups received weekly dietary support. Explicit nutritional and clinical data, as well as calprotectin were collected at baseline and every 2 weeks thereafter. Tolerability was defined as the discontinuation of the intervention for lack of adherence or low adherence judged by weekly interviews and 24 h intake diary. Effectiveness outcomes included clinical remission (i.e., wPCDAI/CDAI), the MINI index that reflects mucosal inflammation (mucosal healing is defined by MINI < 8 points), CRP, ESR, and calprotectin. Those not tolerating either intervention during the first 24 h were excluded; otherwise, both ITT and per‐protocol (PP) analyses are presented.

**Results**: Of the 97 randomized patients, 14 were excluded during the first 24 h and 83 were analyzed, 41 in the T&H arm and 42 in the EEN arm (mean age 14.5 ± 3.7 years). The primary endpoint was met with 36 (88%) patients tolerating T&H versus 22 (52%) tolerating EEN (OR 6.3 [95% CI 2.2–22]; *p* < 0.001).

Week‐8 clinical remission rates were similarly high in both groups whether analyzed by ITT (56% in the T&H vs. 38% in the EEN; *p* = 0.1) or PP (67% vs. 76%; *p* = 0.47). The mean MINI index improved significantly from baseline in both the EEN (14.9 ± 5.6 to 7.1 ± 6.1; *p* < 0.001) and T&H (14.5 ± 4.6 to 8.7 ± 6.4; *p* < 0.001) arms, without a difference across groups (*p* = 0.47). In the ITT analysis, MINI < 8 at week 8 was higher in the T&H arm (54%) than the EEN arm (41%; OR 2.6 [95% CI 1.1–6.3]; *p* = 0.026). Calprotectin decreased significantly from baseline in both arms, without a difference across groups at week‐8 (calprotectin <250 was noted in 38% with T&H vs. 41% with EEN; *p* = 0.84). There were also no differences between EEN and T&H in ESR and CRP, both improving significantly from baseline (both *p* < 0.01).

There were 3 adverse events reported in this trial with at least possible association with the intervention, all mild, 2 in the EEN arm (nausea and dizziness) and 1 in the T&H arm (constipation).

**Conclusion**: T&H is a flexible exclusion diet, with similar effectiveness to EEN in mild–moderate CD and higher tolerance. The diet does not include mandatory foods and does not require PEN.

**Disclosure**: This study was funded by a grant from The Helmsley Charitable Trust.

## LB10 | LENGTHENING USTEKINUMAB DOSING INTERVALS FROM EVERY 8 WEEKS TO EVERY 12 WEEKS IN IBD PATIENTS IN REMISSION: PRELIMINARY RESULTS OF A PROSPECTIVE OBSERVATIONAL COHORT STUDY

M. Devillers ^1^, A. B. Fons^2^, R. West^3^, S. van der Marel^4^, R. Theeuwen^2^, F. Van Schaik^5^, M. Lowenberg^6^, M. C. Visschedijk^7^, M. Pierik^8^, Z. Mujagic^8^, M. Duijvestein^9^, L. Derikx^1^, A. de Vries^1^, A. E. Van Der Meulen ‐ De Jong^2^, Initiative on Crohn and Colitis

^1^ *Gastroenterology and Hepatology, Erasmus MC University Medical Centre, Rotterdam, Netherlands*, ^2^ *Department of Gastroenterology, Leiden University Medical Center, Leiden, Netherlands*, ^3^ *Department of Gastroenterology, Sint Franciscus Gasthuis, Rotterdam, Netherlands*, ^4^ *Gastroenterology, Haaglanden Medisch Centrum, Den Haag, Netherlands*, ^5^ *Department of Gastroenterology, University Medical Centre Utrecht, Utrecht, Netherlands*, ^6^ *Gastroenterology and Hepatology, Amsterdam University Medical Center, Amsterdam, Netherlands*, ^7^ *Gastroenterology and Hematology, University Medical Center, Groningen, Netherlands*, ^8^ *Gastroenterology & Hepatology, Maastricht University Medical Center, Maastricht, Netherlands*, ^9^ *Gastroenterology and Hepatology, Radboudumc, Nijmegen, Netherlands*

**Contact E‐Mail Address**: m.devillers@erasmusmc.nl

**Introduction**: Ustekinumab (UST) is registered as maintenance therapy for patients with inflammatory bowel disease (IBD) in an every 8‐ (Q8W) or 12‐weekly interval (Q12W).^1,2^ Although Q12W is associated with less side effects and reduced treatment costs,^3^ the majority of IBD patients in the Netherlands are on Q8W as maintenance therapy.^4^ The aim of this study is to compare UST drug‐survival between IBD patients in stable remission who lengthen the UST interval from every 8 to every 12 weeks versus those who continue on an 8‐weekly interval.

**Aims & Methods**: IBD patients (aged ≥18 years) who lengthened their UST interval from Q8W to Q12W (Q12W group) were included in the prospective nationwide Initiative on Crohn and Colitis (ICC) Registry between July 2022 and May 2024. Patients were screened on clinical and biochemical remission (Harvey Bradshaw Index ≤5 or Simple Clinical Colitix Activity Index ≤2 and C‐reactive protein ≤10 mg/L and fecal calprotectin ≤250 μg/g) and had to be on a stable Q8W interval for at least 6 months. The interval was lengthened at discretion of the treating physician.

The control group consisted of patients previously included in the registry (March 2016–July 2022) and who were in clinical and biochemical remission on Q8W at least 1 year after initiating UST. Inverse Probability of Treatment Weighting was used to adjust for confounding and selection bias. A drug event was defined as any change in dosage interval or re‐induction during the follow‐up period. The primary outcome was 24‐ and 52‐week cumulative drug survival computed by the Kaplan–Meier method and compared using the log‐rank test.

**Results**: In total, 105 Q12W patients and 88 control patients were included (Table 1). Currently, 93 Q12W patients have completed 24 weeks of follow‐up and 61 patients 52 weeks. 92.4% of the Q12W group versus 96.6% of the control group were previously treated with anti‐TNF therapy and 29.5% versus 34.1% with vedolizumab (*p* = 0.351 and *p* = 0.497, respectively). Cumulative drug survival at 24‐ and 52‐weeks was comparable between the Q12W group and control group (83.8% vs. 88.6% [*p* = 0.370] and 70.2% vs. 76.1% [*p* = 0.451], respectively). The weighted analysis showed no difference in drug survival between the two groups. The median time to drug event was 5.2 months (0.3–11.4). Of the 26 drug events in the Q12W group, one patient stopped UST because of a malignancy, one switched to vedolizumab due to an allergic reaction, two switched to ozanimod due to loss of response, and 24 intensified their UST interval (one to Q10W, 18 to Q8W, one to Q6W and two to Q4W).

**TABLE 1:** Baseline characteristics and outcomes of patients who lengthened their UST interval to every 12 weeks versus those who continued on an 8‐weekly interval.

	Q12W group (*n* = 105)	Control group (*n* = 88)	*p*‐value
Median age, year (IQR)	48.0 (30.0–58.5)	41.0 (31.0–56.0)	0.312
Female, *n* (%)	65 (61.9)	47 (53.4)	0.234
CD, *n* (%)	84 (80.0)	79 (84.5)	0.174
Median disease duration, year (IQR)	15.3 (8.0–25.2)	13.4 (8.1–20.4)	0.341
CD ileocolonic disease localization^a^, *n* (%)	42 (50.0)	41 (51.9)	0.537
UC/IBDU extensive extent^a^, *n* (%)	12 (57.1)	5 (55.6)	0.735
Median UST treatment duration, year (IQR)	2.3 (1.3–3.7)	1.0 (1.0–2.0)	<0.001
Drug events up to 1 year, *n* (%)	26 (24.8)	21 (23.9)	0.885
Reason for drug event, *n* (%)			
Endoscopic/US/MRI disease activity	6 (23.1)	4 (19.0)	
Biochemical disease activity	8 (30.8)	6 (28.6)	
Clinical disease activity	11 (42.3)	6 (28.6)	
Any other reason	1 (3.8)	5 (23.8)	

**Conclusion**: Lengthening the UST interval from every 8 weeks to every 12 weeks appears to be safe and effective in the majority of IBD patients who are in stable remission on an 8 weekly interval. Further research should focus on identifying patients at lowest relapse risk after lengthening UST dosing intervals and the role of UST trough levels.

**References**

1. Feagan BG, Sandborn WJ, Gasink C, Jacobstein D, Lang Y, Friedman JR, et al. Ustekinumab as induction and maintenance therapy for Crohn's disease. *N Engl J Med*. 2016;375(20):1946–1960.
2. Sands BE, Sandborn WJ, Pannaccione R, O'Brien CD, Zhang H, et al. Ustekinumab as induction and maintenance therapy for ulcerative colitis. *N Engl J Med*. 2019;381(13):1201–1214.
3. Bai Y, Sun Y, He Q, Bai X, Yang H. Comparative effectiveness and safety of ustekinumab at different intervals of maintenance phase in inflammatory bowel disease: a systematic review and meta‐analysis. *Eur J Gastroenterol Hepatol*. 2024;36(4):359–370.
4. Straatmijer T, Biemans VBC, Hoentjen F, de Boer NKH, Bodelier AGL, Dijkstra G, et al. Ustekinuma b for Crohn's disease: two‐year results of the Initiative on Crohn and Colitis (ICC) Registry, a nationwide prospective observational cohort study. *J Crohns Colitis*. 2021;15(11):1920–1930.

**Disclosure**: Nothing to disclose.

## From IBS and IBD: Late‐breaking abstracts 08:30–09:30/Strauss 2

## LB11 | CENDAKIMAB EFFICACY AND SAFETY IN ADULT AND ADOLESCENT PATIENTS WITH EOSINOPHILIC ESOPHAGITIS: 24‐WEEK RESULTS FROM THE RANDOMIZED, PLACEBO‐CONTROLLED, PHASE 3 STUDY

A. M. Schoepfer ^1^, C. M. Charriez^2^, S. Zhang^2^, G. Falk^3^, S. Oliva^4^, C. Ma^5^, J. Siffledeen^6^, S. Schroeder^7^, H. Philpott^8^, T. Vanuytsel^9^, C. Contzen^10^, Y. Abe^11^, K. Li^2^, C. L. Zema^2^, A. Venkatasamy^2^, A. Yeshokumar^2^, Y. S. Oh^2^, E. S. Dellon^12^

^1^ *Centre Hospitalier Universitaire Vaudois and University of Lausanne, Lausanne, Switzerland*, ^2^ *Bristol Myers Squibb, Princeton, New Jersey, USA*, ^3^ *Perelman Center for Advanced Medicine, University of Pennsylvania, Bryn Mawr, Pennsylvania, USA*, ^4^ *Azienda Policlinico Umberto I, Rome, Italy*, ^5^ *University of Caglary, Calgary, Alberta, Canada*, ^6^ *South Edmonton Gastroenterology, Edmonton, Alberta, Canada*, ^7^ *Phoenix Children's Hospital, Phoenix, Arizona, USA*, ^8^ *Department of Gastroenterology Lyell McEwin Hospital, Northern Adelaide Local Health Network (NALHN), University of Adelaide, Adelaide, South Australia, Australia*, ^9^ *UZ Leuven, Leuven, Belgium*, ^10^ *AES – DRS – Synexus, Frankfurt, Germany*, ^11^ *Yamagata University Hospital, Yamagata, Japan*, ^12^ *University of North Carolina School of Medicine at Chapel Hill, Chapel Hill, North Carolina, USA*

**Contact E‐Mail Address**: Alain.Schoepfer@chuv.ch

**Introduction**: Eosinophilic esophagitis (EoE) is a chronic, progressive, and immune‐mediated inflammatory disease characterized by esophageal inflammation with infiltration of eosinophils (eos) and symptoms of esophageal dysfunction. Interleukin (IL)‐13, a type 2 inflammatory cytokine, plays a key role in the pathophysiology of EoE. Cendakimab (CEN) is a recombinant, humanized, high‐affinity, and neutralizing monoclonal antibody targeting IL‐13, inhibiting binding to both its receptors, IL‐13Rα1 and IL‐13Rα2. CEN reduced mean esophageal eos counts and improved symptoms and endoscopic features of EoE in a phase 2 study. We report results from a pivotal phase 3 trial (NCT04753697) of CEN in adult and adolescent patients (pts) with EoE after 24 weeks of treatment.

**Aims & Methods**: This multicenter, multinational, randomized, double‐blind, placebo (PBO)‐controlled study enrolled pts aged 12–75 years with active EoE (peak esophageal eos count [PEC] ≥ 15 eos/high power field [hpf] at any two levels of the esophagus), ≥4 dysphagia days (DD) over the 14‐day period prior to day 1 evaluated using the modified Daily Symptom Diary (mDSD), and lack of complete response to ≥8 weeks proton pump inhibitor treatment. The study planned to enroll ∼70% of pts who were steroid inadequate responders or intolerant (steroid IR/I). Pts were randomized 1:1:1 to receive subcutaneous CEN 360 mg weekly for 48 weeks (QW/QW), CEN 360 mg QW for 24 weeks followed by CEN 360 mg every other week for 24 weeks (QW/Q2W), or PBO QW for 48 weeks. Coprimary endpoints were mean change from baseline (BL) to week 24 in DD and proportion of pts with eos histologic response, defined as PEC ≤6/hpf at week 24. Key secondary endpoints included eos histologic response <15/hpf at week 24, endoscopic response (mean change in Endoscopic Reference Score [EREFS] total score), mean change in EoE Histology Scoring System (EoEHSS) grade/stage score, and mean change in mDSD composite score, from BL to week 24. Safety was also evaluated.

**Results**: Overall, 430 pts (∼66% steroid IR/I) were randomized to CEN (*n* = 286; QW/QW, *n* = 143, QW/Q2W, *n* = 143), or PBO (*n* = 144). In pts treated with CEN 360 mg QW, there was a statistically significant reduction from BL to week 24 in DD versus PBO (−6.1 vs. −4.2 days; difference [95% CI] vs. PBO: −1.9 [−3.0 to −0.8]; *p* = 0.0005) and a statistically significantly higher proportion of pts with eos histologic response at week 24 versus PBO (28.6% vs. 2.2%; difference [95% CI] vs. PBO: 26.4 [20.6 to 32.2]; *p* < 0.0001) (Table). Statistically significant improvements in all key secondary endpoints were observed for CEN‐treated versus PBO‐treated pts (Table). At week 24, adverse events (AE) related to study drug occurred in 30.3% and 18.9% of pts in the CEN and PBO arms, with AEs leading to discontinuation in 1.4% and 0.7% of pts, respectively. No deaths occurred.

**Table:** TABLE Efficacy results at week 24.

	Treatment	CEN 360 mg QW (*n* = 286)	PBO QW (*n* = 144)	Difference (95% CI); *p* value
Coprimary endpoints	LS mean change from baseline in DD	−6.1	−4.2	−1.9 (−3.0 to −0.8); *p* = 0.0005
	Patients with eos histologic response (≤6/hpf), %	28.6	2.2	26.4 (20.6–32.2); *p* < 0.0001
Key secondary endpoints	Patients with eos histologic response (<15/hpf), %	43.1	3.1	40.0 (33.3–46.7); *p* < 0.0001
	LS mean change in EREFS^a^ total score	−5.2	−1.2	−4.0 (−4.8 to −3.2); *p* < 0.0001
	LS mean change in EoEHSS grade score	−26.2	−3.7	−22.5 (−25.5 to −19.5); *p* < 0.0001
	LS mean change in EoEHSS stage score	−31.7	−6.3	−25.4 (−28.9 to −21.9); *p* < 0.0001
	LS mean change in mDSD composite score	−12.8	−8.7	−4.1 (−6.4 to −1.8); *p* = 0.0005

Abbreviation: LS, least squares.

^a^EREFS is a validated approach for assessing the presence and severity of the major endoscopic features of EoE. EREFS were scored on a 0–8 point scale (edema [0–1], rings [0–3], exudates [0–2], furrows [0–1], and stricture [0–1]) at 3 levels of the esophagus (proximal, mid, and distal), with total score range of 0–24.

**Conclusion**: Treatment with CEN demonstrated statistically significant improvement in symptoms and histologic and endoscopic features of EoE versus PBO. CEN was generally safe and well‐tolerated through week 24.

**Disclosure**: AS has served a consultant for Adare/Ellodi, AbbVie, AstraZeneca, Celgene/Receptos/Bristol Myers Squibb, Dr. Falk Pharma, Gossamer Bio, GSK, Janssen, MSD, Pfizer, Regeneron/Sanofi, Takeda, and Vifor; and has received grant/research support from Adare/Ellodi, Celgene/Receptos/Bristol Myers Squibb, GSK, and Regeneron/Sanofi. CMC, SZ, KL, AV, AY, and YSO are employees and shareholders of Bristol Myers Squibb. CLZ is a contractor to Bristol Myers Squibb. ESD has served as a consultant for Abbott, AbbVie, Adare/Ellodi, Aimmune, Akesobio, Alfasigma, ALK, Allakos, Amgen, Apollo, Aqilion, Arena/Pfizer, Aslan, AstraZeneca, Avir, Biorasi, Bryn, Calypso, Celgene/Receptos/Bristol Myers Squibb, Celldex, Eli Lilly, EsoCap, Eupraxia, Dr. Falk Pharma, Ferring, GSK, Gossamer Bio, Holoclara, Invea, Knightpoint, Landos, LucidDx, Morphic, Nexstone Immunology/Uniquity, Nutricia, Parexel/Calyx, Phathom, Regeneron, Revolo, Robarts/Alimentiv, Salix, Sanofi, Shire/Takeda, Target RWE, and Upstream Bio; has received grant/research support from Adare/Ellodi, Allakos, Arena/Pfizer, AstraZeneca, Eupraxia, GSK, Meritage, Miraca, Nutricia, Celgene/Receptos/Bristol Myers Squibb, Regeneron, Revolov, and Shire/Takeda; and has received educational grants from Allakos, Aqilion, Holoclara, and Invea. GF has served as a consultant for Adare/Ellodi, Allakos, Celgene/Receptos/Bristol Myers Squibb, LucidDx, Nexstone Immunology/Uniquity, Phathom, Regeneron/Sanofi, Shire/Takeda, and Upstream Bio; has received grant/research support from Adare/Ellodi, Allakos, Arena/Pfizer, Celldex, Celgene/Receptos/Bristol Myers Squibb, Regeneron, Sanofi, Revolov, and Shire/Takeda. CM has received consulting fees from AbbVie, Alimentiv, Amgen, AVIR Pharma Inc., Bristol Myers Squibb, Celltrion, Eli Lilly, Ferring, Fresenius Kabi, Janssen, McKesson, Mylan, Pendopharm, Pfizer, Prometheus Biosciences Inc., Roche, Sanofi, Takeda, Tillotts Pharma; speaker's fees from AbbVie, Amgen, AVIR Pharma Inc., Alimentiv, Bristol Myers Squibb, Eli Lilly, Ferring, Fresenius Kabi, Janssen, Organon, Pendopharm, Pfizer, Sanofi, Takeda, Tillotts Pharma; royalties from Springer Publishing; research support from AbbVie, Ferring, Pfizer. HP has served as a consultant for Dr. Falk Pharma, Arena/Pfizer, GSK and Abbott. TV is a speaker for Dr. Falk Pharma and Bristol Myers Squibb; consultancy for Dr. Falk Pharma, Bristol Myers Squibb; research grant from Dr. Falk Pharma. SO has served a consultant for Dr. Falk Pharma, Medtronic, Regeneron/Sanofi, Celgene/Receptos/Bristol Myers Squibb; and has received grant/research support from Medtronic, Regeneron/Sanofi, Alfasigma.

## LB12 | RCT ASSESSING EFFECT OF 6‐WEEK INTERVENTION WITH 10GR DAILY DOSE OF PARTIALLY HYDROLYZED GUAR GUM (PHGG) ON SPONTANEOUS BOWEL MOVEMENT FREQUENCY IN ADULTS WITH CHRONIC CONSTIPATION

M. J. Buckley ^1^, C. d'Urzo^2^, I. Aouadi^2^, J. Benyacoub^2^, S. Delgado‐Aros^2^

^1^ *Department of Gastroenterology, Mercy University Hospital, Cork, Ireland*, ^2^ *Global Research and Clinical Development, Société des Produits Nestlé SA, Nestlé Health Sciences, Vevey, Switzerland*

**Contact E‐Mail Address**: drmjmbuckley@gmail.com

**Introduction**: Non‐absorbable fiber supplements are widely used as a first‐line treatment for constipation despite lack of consistent evidence. Two reviews identified placebo‐controlled randomized trials evaluating soluble fibers (psyllium [*n* = 3] maltodextrin [*N* = 1] and inulin [*N* = 1]) providing moderate evidence favoring their use in constipated subjects.

Partially Hydrolyzed Guar Gum (PHGG) is water‐soluble fiber derived from the endosperm of Cyamopsis tetragonolobus L seeds. There are series of small to medium studies assessing 1–4 weeks intervention with PHGG in various population with constipation. These data suggest a dose‐effect with an optimal dose of approximately 10 g. Furthermore, the PHGG benefit was observed as soon as 1 week. However, data suggest that longer duration of treatment may bring added benefit, with more subjects achieving response.

**Aims & Methods**: To evaluate safety and efficacy of 6 weeks of daily consumption of 10gr of PHGG (SunfiberTM) as compared to placebo in adults with functional‐ or IBS‐constipation. This was a double‐blind, placebo‐controlled, randomized, 2‐arm, and parallel design study. Constipation outcomes measures were collected through daily eDiary and included: (1) Bowel movement (BM) frequency and characteristics, (2) Bristol stool scale (BSS), and (3) PAC‐SYM questionnaire. The primary efficacy endpoint was change in weekly mean frequency of spontaneous BM (SBM). Response rate was assessed as exploratory endpoint and responder was defined as having ≥3 SBM/week and an increase of ≥1 SBM from baseline for at least 4 of the 6 weeks of treatment. Treatment groups were compared using ANCOVA model, which included baseline, fiber intake and age as covariates. Data reported are ITT mean and percentage (95% CI) unadjusted estimates. Adjusted ANCOVA model *p* values are reported for ITT and PP populations.

**Results**: Patients (*N* = 160) were randomized to receive PHGG (*N* = 80) or pbo (*N* = 80). Both groups were comparable at baseline (Table 1) SBM was significantly increased after PHGG versus pbo; *p* = 0.033 (ITT) & 0.029 (PP). As per entry criteria all subjects had <3 SBM/week at baseline. After treatment 40/71 (50%) and 26/73 (32.5%) reached SBM ≥3 in the active and pbo group, respectively. Pre‐defined responder definition, ≥3 SBM/week and an increase of ≥1 SBM from baseline for at least 4 of the 6 weeks of treatment was achieved by 34.2% (27%; 48.5%) versus 17.7% (12%; 30%); *p* = 0.018 (ITT) & 0.019 (PP). BSS increased on average 1 (0.8; 1.2) and 0.8 (0.5; 1.0) points from baseline to the end of treatment in active and pbo, respectively; *p* = 0.149 (ITT) & 0.057 (PP). PAC‐SYM decreased 0.85 (0.70; 0.99) and 0.69 (0.54; 0.84) in the active and pbo groups, respectively; *p* = 0.049 (ITT) & 0.0621 (PP). There were no SAEs and no discontinuations due to AEs. 30 (37.5%) and 34 (42.5%) patients reported some AEs in the pbo and PHGG groups, respectively. Overall, PHGG was well tolerated (Table 2).

**TABLE 1:** Demographic and baseline characteristics.

Baseline mean (95% CI)	Placebo (*N* = 80)	PHGG (*N* = 80)
Female (*n*, %)	70 (87.5%)	66 (82.5%)
Age (years)	34 (31.6; 36.5)	36 (33.4; 38.8)
Fiber intake (g/day)	15.50 (14.3, 16.7)	14.63 (13.6; 15.6)
SBM	1.52 (1.42; 1.62)	1.45 (1.35; 1.55)
Bristol stool scale (BSS)	2.10 (1.92; 2.29)	2.05 (1.85; 2.26)
PAC‐SYM total score	1.85 (1.69; 2.01)	1.85 (1.70; 1.99)

**TABLE 2:** Safety.

No. subjects (%)		
Abdominal pain/discomfort (mild)	4 (5%)	3 (4%)
Diarrhea	3 (4%)	2 (2.5%)

**Conclusion**: PHGG is well tolerated and increases the frequency of spontaneous bowel movements without increasing GI symptoms in patients with chronic functional or IBS‐constipation.

**References**

Müller‐Lissner S, Tack J, Feng Y, Schenck F, Specht Gryp R. Levels of satisfaction with current chronic constipation treatment options in Europe–an internet survey. *Aliment Pharmacol Ther*. 2013;37(1):137–145.

Ramkumar D, Rao SS. Efficacy and safety of traditional medical therapies for chronic constipation: systematic review. *Am J Gastroenterol*. 2005;100(4):936–971.

Suares NC, Ford AC. Prevalence of, and risk factors for, chronic idiopathic constipation in the community: systematic review and meta‐analysis. *Am J Gastroenterol*. 2011;106(9):1582–1591.

Shaheen NJ, Hansen RA, Morgan DR, Gangarosa LM, Ringel Y, Thiny MT, et al. The burden of gastrointestinal and liver diseases, 2006. *Am J Gastroenterol*. 2006;101(9):2128–2138.

Shah ND, Chitkara DK, Locke GR, Meek PD, Talley NJ. Ambulatory care for constipation in the United States, 1993–2004. *Am J Gastroenterol*. 2008;103(7):1746–1753.

Kapoor MP, Sugita M, Fukuzawa Y, Okubo T. Impact of partially hydrolyzed guar gum (PHGG) on constipation prevention: A systematic review and meta‐analysis. *J Funct Foods*. 2017;33:52–66.

**Disclosure**: Nothing to disclose.

## LB13 | MOTION SERIFOS: TRADIPITANT EFFECTIVE IN THE PREVENTION OF VOMITING IN MOTION SICKNESS DURING SEA TRAVEL

V. Polymeropoulos ^1^, M. Bushman^1^, L. Kiely^1^, T. Davis^1^, A. Giles^1^, D. Morgan^1^, R. Mourad^1^, C. Xiao^1^, C. Polymeropoulos^1^, G. Birznieks^1^, M. Polymeropoulos^1^

^1^ *Vanda Pharmaceuticals, Washington, District of Columbia, USA*

**Contact E‐Mail Address**: vasilios.polymeropoulos@vandapharma.com

**Introduction**: Motion sickness has altered the course of history, including afflicting the sailors in the Battle of the Red Cliffs, potentially leading to the fall of the Han Dynasty. Motion sickness remains a significant challenge for both professional and leisure travelers, including during spaceflight where motion sickness affects 60%–80% of astronauts. Neurokinin‐1 receptors (NK1) are widely expressed in the body with important locations in the emetic pathway in the network of brainstem nuclei and the gut. In the intestine, NK1 receptors are widely distributed, regulating motility, pain, and inflammation. Tradipitant is a novel NK1 receptor antagonist being studied for the treatment of motion sickness. In two previous studies in the coastal waters of the United States with 491 total participants, tradipitant significantly prevented vomiting as compared to placebo. In this study we sought to further understand the effects of tradipitant 170 mg and tradipitant 85 mg in motion sickness.

**Aims & Methods**: The Motion Serifos study was a multicenter, randomized, double‐blind, and placebo‐controlled study where 316 participants embarked on boat trips under varied sea conditions and received 1:1:1 tradipitant 170 mg, tradipitant 85 mg, or placebo. Participants evaluated their symptoms at 30 min intervals during the approximately 4 h expedition, with questionnaires evaluating instances of vomiting and severity of nausea.

**Results**: Both dosing arms of tradipitant (170 and 85 mg) were superior to placebo in the prevention of vomiting (*p* = 0.000002, *p* = 0.0014). Tradipitant was superior to placebo in the prevention of severe nausea and vomiting across varied sea conditions (*p* = 0.00003).

**Conclusion**: Tradipitant could be of significant utility for motion sickness affected travelers in both civilian and military settings across air, land, and sea. Given the adverse effects and incomplete efficacy of currently available therapies, there remains a need for a novel efficacious therapeutic with a desirable tolerability profile.

**Disclosure**: All authors are employees of Vanda Pharmaceuticals. The study was sponsored by Vanda Pharmaceuticals.

## LB14 | EFFICACY AND SAFETY OF TAMUZIMOD IN MODERATELY TO SEVERELY ACTIVE ULCERATIVE COLITIS THROUGH 52 WEEKS: PHASE 2 LONG‐TERM EXTENSION DATA

S. Danese ^1^, R. Panaccione^2^, G. R. D'Haens^3^, S. Schreiber^4^, V. Jairath^5^, A. DuVall^6^, J. Kierkuś^7^, S. Naik^8^, K. Gilder^8^, B. Lindstrom^8^, W. J. Sandborn^9^, S. Vermeire^10^, D. T. Rubin^11^, L. Peyrin‐Biroulet^12^, B. E. Sands^13^

^1^ *Department of Gastroenterology and Endoscopy, Vita‐Salute San Raffaele University ‐ IRCCS San Raffaele Scientific Institute, Milan, Italy*, ^2^ *Inflammatory Bowel Disease Unit, Division of Gastroenterology and Hepatology, University of Calgary, Calgary, Alberta, Canada*, ^3^ *Department of Gastroenterology and Hepatology, Amsterdam University Medical Centres, Amsterdam, Netherlands*, ^4^ *Department of Internal Medicine I, University Hospital Schleswig‐Holstein, Kiel, Germany*, ^5^ *Division of Gastroenterology, Department of Medicine, Western University, London, Ontario, Canada*, ^6^ *Tyler Research Institute, Tyler, Texas, USA*, ^7^ *Department of Gastroenterology, Hepatology, Nutritional Disorders and Paediatrics, Children's Memorial Health Institute, Warsaw, Poland*, ^8^ *Ventyx Biosciences, Inc., San Diego, California, USA*, ^9^ *Division of Gastroenterology, University of California, San Diego, California, USA*, ^10^ *Department of Gastroenterology & Hepatology, University Hospital Leuven, Leuven, Belgium*, ^11^ *Inflammatory Bowel Disease Center, University of Chicago Medicine, Chicago, Illinois, USA*, ^12^ *Department of Gastroenterology, Inserm, NGERE, University of Lorraine, Vandoeuvre‐les‐Nancy, France*, ^13^ *Dr Henry D Janowitz Division of Gastroenterology, Icahn School of Medicine at Mount Sinai, New York, New York, USA*

**Contact E‐Mail Address**: sdanese@hotmail.com

**Introduction**: Tamuzimod is a novel, oral, and selective sphingosine‐1‐phosphate‐1 receptor modulator in development for the treatment of ulcerative colitis (UC). The efficacy and safety of tamuzimod as induction therapy was previously demonstrated in a phase 2, multicenter, randomized, double‐blind, and placebo‐controlled trial (NCT05156125).^1^ Here we report 52‐week maintenance data from the long‐term extension (LTE) treatment period of that trial.

**Aims & Methods**: Adults (*N* = 213) with a modified Mayo score of 4–9 and inadequate/loss of response/intolerance to at least one approved UC therapy were randomized 1:1:1 to once‐daily treatment with oral tamuzimod (30 or 60 mg) or placebo for 13 weeks. Patients who completed the 13‐week induction treatment period and achieved clinical response were eligible for the LTE treatment period and once daily blinded maintenance therapy with their previously assigned treatment for up to 39 weeks. Clinical, endoscopic, and histologic outcomes (defined in Table) were evaluated at week 52. Adverse events (AEs) and laboratory abnormalities were also assessed for safety.

**Results**: Of 203 patients who completed week 13, 85 were eligible and entered the LTE treatment period (22/66 placebo, 34/70 tamuzimod 30 mg, 29/67 tamuzimod 60 mg). At week 52, clinical remission occurred in 50.0% of patients who received maintenance treatment with either tamuzimod 30 mg (16/32) or 60 mg (14/28) compared to 18.2% (4/22) of patients who received placebo. Endoscopic remission occurred in 43.8% (14/32) and 46.4% (13/28) of patients who received tamuzimod 30 and 60 mg, respectively, compared to 18.2% (4/22) of patients who received placebo. Higher rates of sustained (week 13 and week 52) clinical remission, symptomatic remission, endoscopic improvement, and the composite outcomes of endoscopic and clinical or histologic remission, and endoscopic improvement and histologic remission were also observed with both tamuzimod doses compared to placebo (see Table). Consistent with these efficacy outcomes and tamuzimod's proposed mechanism of action, absolute lymphocyte count (ALC) was relatively unchanged from baseline in patients who received placebo, whereas sustained reductions in ALC were observed for both tamuzimod doses over time, with a 58.9% and 71.5% decrease in ALC from baseline observed at week 52 for patients who received tamuzimod 30 and 60 mg, respectively. No new safety signals were identified during the LTE treatment period. No instances of bradycardia, atrioventricular block, serious infections, or macular edema were observed and no deaths occurred.

**Table:** TABLE Efficacy outcomes at Week 52.

Outcome	Placebo (*N* = 22)	Tamuzimod 30 mg (*N* = 32)	Difference from placebo (*p*‐value)^a^	Tamuzimod 60 mg (*N* = 28)	Difference from placebo (*p*‐value)^b^
Clinical remission^c^, *n* (%)	4 (18.2)	16 (50.0)	31.8 (0.0078)	14 (50.0)	31.8 (0.0206)
Sustained clinical remission^d^, *n* (%)	3 (13.6)	10 (31.3)	17.6 (0.1236)	9 (32.1)	18.5 (0.2751)
Symptomatic remission^e^, *n* (%)	9 (40.9)	22 (68.8)	27.8 (0.0137)	21 (75.0)	34.1 (0.0102)
Endoscopic improvement^f^, *n* (%)	6 (27.3)	16 (50.0)	22.7 (0.0508)	17 (60.7)	33.4 (0.0237)
Endoscopic remission^g^, *n* (%)	4 (18.2)	14 (43.8)	25.6 (0.0315)	13 (46.4)	28.2 (0.0757)
Endoscopic and clinical remission^h^, *n* (%)	3 (13.6)	14 (43.8)	30.1 (0.0117)	11 (39.3)	25.6 (0.0872)
Endoscopic and histologic remission^i^, *n* (%)	2 (9.1)	12 (37.5)	28.4 (0.0193)	7 (25.0)	15.9 (0.1759)
Endoscopic improvement‐histologic remission^j^, *n* (%)	3 (13.6)	12 (37.5)	23.9 (0.0462)	8 (28.6)	14.9 (0.2128)

Abbreviations: ES, endoscopic subscore; *N*, number participants in the LTE Full Analysis Set who were eligible for LTE and had a baseline MMS of 5–9; NRI, nonresponder imputation; RB, rectal bleeding; and SF, stool frequency.

^a^ Percent difference between placebo and tamuzimod 30 mg (NRI); nominal *p*‐value based on a two‐sided Cochran–Mantel–Haenszel (CMH) test.

^b^ Percent difference between placebo and tamuzimod 60 mg (NRI); nominal *p*‐value based on a two‐sided CMH test.

^c^ Modified Mayo SF subscore ≤1, RB subscore = 0, and ES ≤ 1 (excluding friability).

^d^ Clinical remission at week 13 and 52.

^e^ Modified Mayo SF subscore ≤1 and RB subscore = 0.

^f^ Modified Mayo ES ≤ 1 (excluding friability).

^g^ Modified Mayo ES = 0.

^h^ Modified Mayo SF subscore ≤1, RB subscore = 0, and ES = 0.

^i^ Modified Mayo ES = 0 and Geboes Index score <2.0.

^j^ Modified Mayo ES ≤ 1 (excluding friability) and Geboes Index score <2.0.

**Conclusion**: Maintenance treatment with both 30 and 60 mg tamuzimod was efficacious and well‐tolerated for up to 52 weeks. High rates of both clinical and endoscopic remission observed during tamuzimod induction and maintenance therapy may be potentially due to rapid and sustained ALC reductions. Efficacy and safety data from this LTE treatment period support the continued clinical development of tamuzimod for treatment of UC.

**Reference**

1. Sands BE, Panaccione R, D'Haens G, Schreiber S, DuVall A, Kierkus J, et al. Efficacy and safety of the oral selective sphingosine‐1‐phosphate‐1 receptor modulator VTX002 in moderately to severely active ulcerative colitis: Results from a randomized, double‐blind, placebo‐controlled, phase 2 trial. *Gastroenterology*. 2024;166:S‐240. https://doi.org/10.1016/S0016‐5085(24)01036‐9

**Disclosure**: SD: consulting for AbbVie, Alimentiv, Allergan, Amgen, Applied Molecular Transport, AstraZeneca, Athos Therapeutics, Biogen, Boehringer Ingelheim, Bristol Myers Squibb, Celgene, Celltrion, DrFalk Pharma, Eli Lilly, Enthera, Ferring, Gilead, Hospira, Inotrem, Janssen, J&J, Morphic, MSD, Mundipharma, Mylan, Pfizer, Roche, Sandoz, Sublimity Therapeutics, Takeda, Teladoc Health, TiGenix, UCB, Vial, and Vifor and lecture fees from Abbvie, Amgen, Ferring, Gilead, Janssen, Mylan, Pfizer, and Takeda.

RP: consulting for Abbott, AbbVie, Abbivax, Alimentiv, Amgen, AnaptysBio, Arena Pharmaceuticals, AstraZeneca, Biogen, Boehringer Ingelheim, Bristol‐Myers Squibb, Celgene, Celltrion, Cosmos Pharmaceuticals, Eisai, Elan, Eli Lilly, Ferring, Galapagos, Fresenius Kabi, Genentech, Gilead, Glaxo‐Smith Kline, JAMP Bio, Janssen, Merck, Mylan, Novartis, Oppilan Pharma, Organon, Pandion Pharma, Pendopharm, Pfizer, Progenity, Prometheus Biosciences, Protagonist Therapeutics, Roche, Sandoz, Satisfai Health, Shire, Sublimity Therapeutics, Spyre Therapeutics, Takeda, Theravance Biopharma, Trellus, Union Biopharma, Viatris, Ventyx, and UCB; speaker for AbbVie, Amgen, Arena Pharmaceuticals, Bristol‐Myers Squibb, Celgene, Eli Lilly, Ferring, Fresenius Kabi, Gilead, Janssen, Merck, Organon, Pfizer, Roche, Sandoz Shire, and Takeda; and advisory boards for: AbbVie, Alimentiv, Amgen, Arena Pharmaceuticals, AstraZeneca, Biogen, Boehringer Ingelheim, Bristol‐Myers Squibb, Celgene, Eli Lilly, Ferring, Fresenius Kabi, Genentech, Gilead, Glaxo‐Smith Kline, JAMP Bio, Janssen, Merck, Mylan, Novartis, Oppilan Pharma Organon, Pandion Pharma, Pfizer, Progenity, Protagonist Therapeutics, Roche, SandozShire, Sublimity Therapeutics, Takeda, and Ventyx. GRD: advisor fees from AbbVie, Alimentiv, AstraZeneca, Boehringer Ingelheim, Bristol Myers Squibb, Celltrion, Eli Lilly and Company, GlaxoSmithKline, Pfizer Inc, Johnson and Johnson, Takeda Pharmaceutical Company, Prometheus Biosciences, and Ventyx Biopharma. SS: Personal fees were received from Abbvie, Amgen, Arena, Biogen, Bristol Meyers Squibb, Celgene, Celltrion, Falk, Ferring, Fresenius Kabi, Galapagos, Gilead, IMAB, Janssen, Lilly, Morphic, MSD, Mylan, Novartis, Pfizer, Protagonist, Provention Bio, Roche, Sandoz/Hexal, Shire, Takeda, Theravance, and Ventyx. VJ: consulting/advisory board for AbbVie, Alimentiv, Arena Pharmaceuticals, Asahi Kasei Pharma, Asieris, Astra Zeneca, Avoro Capital, Bristol Myers Squibb, Celltrion, Eli Lilly, Endpoint Health, Enthera, Ferring, Flagship Pioneering, Fresenius Kabi, Galapagos, Gilde Healthcare, GlaxoSmithKline, Genentech, Gilead, Innomar, JAMP, Janssen, Merck, Metacrine, Mylan, MRM Health, Pandion, Pendopharm, Pfizer, Protagonist, Prometheus Biosciences, Reistone Biopharma, Roche, Roivant, Sandoz, Second Genome, Sorriso, Synedgen, Takeda, TD Securities, Teva, Topivert, Ventyx, and Vividion and speaker for Abbvie, Ferring, Bristol Myers Squibb, Galapagos, Janssen Pfizer Shire, Takeda, and Fresenius Kabi. AD: nothing to disclose. JK: consulting/speaker/advisory board for Lilly, BMS, Nestle, and Danon. SN, KG, BL: employees of Ventyx Biosciences. WJS: consulting for Alimentiv and Shoreline Biosciences; stock/stock options from Prometheus Biosciences, Prometheus Laboratories, Shoreline Biosciences, Ventyx, Mirador Therapeutics; employee at Ventyx and Mirador Therapeutics; and serving on the Board of Directors at Prometheus Laboratories. SV: financial research support from AbbVie, J&J, Pfizer, Takeda and Galapagos and speaker/consulting fees from AbbVie, Abivax, Abolerls Pharma, AgomAb, Alimentiv, Arena Pharmaceuticals, Astrazeneca, Biora Therapeutics, BMS, Boehringer Ingelheim, Celgene, Cytoki Pharma, Dr Falk Pharma, Ferring, Galapagos, Genentech Roche, Gilead, GSK, Hospira, lmidomics, Janssen, J&J, Lilly, Materia Prima, Mestag Therapeutics, Microbiotica, MiroBio, Morphic, MrMHealth, Mundipharma, MSD, Pfizer, Prodigest, Progenity, Prometheus, Robarts Clinical Trials, Surrozen, Takeda, Theravance, Tillots Pharma AG, VectivBio, Ventyx, and Zealand Pharma. DTR: grants from Takeda; consulting for Abbvie, Altrubio, Apex, Avalo Therapeutics, Bristol‐Myers Squibb, Buhlmann Diagnostics Corp, Celgene, Connect BioPharma, Intouch Group, Iterative Health, Janssen Pharmaceuticals, Lilly, Pfizer, Samsung Neurologica, and Takeda; and serving on Board of Trustees for Crohn's & Colitis Foundation and Board of Directors for Cornerstones Health. LP: consulting for Abbvie, Abivax, Adacyte, Alimentiv, Amgen, Applied Molecular Transport, Arena, Banook, Biogen, BMS, Celltrion, Connect Biopharm, Cytoki Pharma, Enthera, Ferring, Fresenius Kabi, Galapagos, Genentech, Gilead, Gossamer Bio, GSK, IAC Image Analysis, Index Pharmaceuticals, Inotrem, Janssen, Lilly, Medac, Mopac, Morphic, MSD, Nordic Pharma, Novartis, Oncodesign Precision Medicine, ONO Pharma, OSE Immunotherapeuthics, Pandion Therapeuthics, Par' Immune, Pfizer, Prometheus, Protagonist, Roche, Samsung, Sandoz, Sanofi, Satisfay, Takeda, Telavant, Theravance, Thermo Fischer, Tigenix, Tillots, Viatris, Vectivbio, Ventyx, and Ysopia; grants from Celltrion, Fresenius Kabi, Medac, MSD, and Takeda; and lecture fees from Abbvie, Amgen, Arena, Biogen, Celltrion, Ferring, Galapagos, Genentech, Gilead, Janssen, Lilly, Medac, MSD, Nordic Pharma, Pfizer, Sandoz, Takeda, Tillots, and Viatris. BES: non‐financial support/personal fees/stock/stock options from Ventyx Biosciences during the conduct of the study and grant/personal fees/non‐financial support from AbbVie, Abivax, Adiso Therapeutics, Agomab, Alimentiv, Amgen, AnaptysBio, Arena Pharmaceuticals, Artugen Pharmaceuticals, AstraZeneca, Biora Therapeutics, Boehringer Ingelheim, Boston Pharmaceuticals, Bristol Myers Squibb, Calibr, Celgene, Celltrion, ClostraBio, Equilium, Enthera, Evommune, Ferring, Fresenius Kabi, Galapagos, Genentech, Gilead Sciences, Glaxo SmithKline, GossamerBio, Index Pharmaceuticals, Innovation Pharmaceuticals, Inotrem, Janssen, J&J, Kalleido, Kallyope, Lilly, Merck, Microbiotica, Mobius Care, Morphic Therapeutics, MRM Health, Nexus Therapeutics, Immunyx Therapeutics, Nimbus Discovery, Odyssey Therapeutics, Pfizer, Progenity, Prometheus Biosciences, Prometheus Laboratories, Protagonist Therapeutics, Q32 Bio, Rasayana Therapeutics, Recludix Therapeutics, Reistone Biotherapeutics, Sun Pharma, Surrozen, Target RWE, Takeda, Teva, Theravance Biopharma, TLL Pharmaceutical, TR1X, Union Therapeutics, Palisade Bio, and outside submitted work.

## LB15 | YIELD OF COLONOSCOPY IN EVALUATION YOUNG WOMEN WITH CONSTIPATION

A. Beshara^1^, N. Abu Freha ^2^

^1^ *Gastroenterology, Hillel Yaffe Medical Center, Hadera, Israel*, ^2^ *Soroka Medical Center, Beer Sheva, Israel*

**Contact E‐Mail Address**: abufreha@yahoo.de

**Introduction**: Constipation is one the most common gastrointestinal disorders among women. Causing factors are various. We aimed to assess the role of colonoscopy in evaluation young women with constipation.

**Aims & Methods**: A multi‐center, large cohort, and cross‐sectional retrospective study included all colonoscopies performed between 2016 and 2021 in seven endoscopy departments. The indications and findings of the procedures were collected and findings of young women aged 40 years and younger with constipation as indication were compared to older women.

**Results**: A total cohort of 377,795 patients, 198,629 (52.6%) females and 179,166 (47.4%) males. 7872 females underwent colonoscopy for constipation and other indications **(Cohort 1)**. 6852 women referred to colonoscopy for constipation only **(Cohort 2)**. Women younger than 50 years had more associated symptoms than older women. 75% of colonoscopies in women <40 years were normal in both cohorts. In **Cohort 1**, inflammatory bowel diseases (IBD) was significantly higher in young women <40 years as compared with ulcerative colitis (UC) 12 (1.2%) and Crohn's 7 (0.7%) *p* < 0.001. In **Cohort 2**, among women <40 years, UC was found in 6 (0.7%) *p* = 0.01 and Crohn's in 2 (0.2%) *p* = 0.05. In both cohorts, diverticulosis and polyps rates exponentially increased with age. The increase was remarkably noticed at ages >40 years in comparison to younger subjects 18–39 years, *p* < 0.001. In **Cohort 1**, diverticulosis and polyps rates among young women <40 years was 0.5% and 7.4%, respectively. In **Cohort 2** diverticulosis rate 0.4% and polyps 8%. One case (0.1%) of colorectal cancer (CRC) in <40 years women and 0.3% in women aged 40–49 years. In comparison between two genders: **Cohort 1** similar prevalence of IBD found in males and females in all ages (UC *p* = 0.94, Crohn's *p* = 0.67). Higher rate of diverticulosis among males in all ages *p* < 0.05. Higher rate of polyps in all ages including young males <40 years, *p* = 0.001. In **Cohort 2** no significant difference in the prevalence of IBD (*p* > 0.05), diverticulosis rate (*p* = 0.15), polyps rate (*p* = 0.34), and CRC rate (*p* = 0.5) among young pts <40 years of both genders.

**Table:** TABLE 1

6852 females	Age group	Age group	Age group	Age	*p* value all groups	*p* Group 1 versus 2	*p* Groups 1 versus 3	*p* Group 1 versus 4	*p* Group 1 versus 2 + 3 + 4
	18–39 years (1)	40–49 (2)	50–59 years (3)	61 years and older (4)					
	*n* = 807	*n* = 1328	*n* = 1739	*n* = 2978					
Findings in colonoscopy									
UC	6 (0.7%)	4 (0.3%)	2 (0.1%)	4 (0.1%)	0.008	0.147	0.008	0.003	0.001
CD	2 (0.2%)	3 (0.2%)	0	0	0.011	0.919	0.038	0.007	0.050
Angiodysplasia	0	0	2 (0.1%)	3 (0.1%)	0.513	0	0.335	0.367	0.414
Diverticulosis	3 (0.4%)	35 (2.6%)	132 (7.6%)	614 (20.6%)	<0.001	<0.001	<0.001	<0.001	<0.001
Polyp of colon	65 (8.1%)	217 (16.3%)	396 (22.8%)	998 (33.5%)	<0.001	<0.001	<0.001	<0.001	<0.001
CRC	1 (0.1%)	4 (0.3%)	3 (0.2%)	11 (0.4%)	0.509	0.411	0.773	0.271	0.378
Normal colonoscopy	608 (75.3%)	863 (65%)	972 (56%)	1170 (39.3%)	<0.001	<0.001	<0.001	<0.001	<0.001

**Conclusion**: The yield of colonoscopy in investigation isolated constipation in young females <40 years is not of a considerable diagnostic value. Clinical understanding should guide each case manager by using the correct beneficial diagnostic tools avoiding unnecessary invasive procedures.

**Disclosure**: Nothing to disclose.

## Between liver and pancreas—Game on: Late‐breaking abstracts 14:30–15:30/A1

## LB16 | ACCELERATED VS STEP‐UP ENDOSCOPIC TREATMENT FOR LARGE PANCREATIC WALLED‐OFF NECROSIS: INTERIM ANALYSIS OF A SINGLE‐CENTER RANDOMIZED TRIAL (ACCELERATE TRIAL)

G. A. Olsen ^1^, P. N. Schmidt^1^, A. Hadi^1^, A. P. Prahm^1^, M. P. Werge^1^, d. F. Schefte^1^, M. L. Lauritsen^1,2^, E. F. Hansen^1^, S. Novovic^1,2^, J. G. Karstensen^1,2^

^1^ *Pancreatitis Centre East, Gastro Unit, Copenhagen University Hospital ‐ Amager and Hvidovre, Hvidovre, Denmark*, ^2^ *Department of Clinical Medicine, University of Copenhagen, Copenhagen, Denmark*

**Contact E‐Mail Address**: gitte.aabye.olsen@regionh.dk

**Introduction**: Pancreatic walled‐off necrosis (WON) is a complication of acute necrotizing pancreatitis often resulting in prolonged hospitalization.^1^ While the endoscopic step‐up approach with endoscopic ultrasound guided transmural drainage followed by, if necessary, endoscopic necrosectomy, has become gold standard for treating WON, a recent study suggests that performing necrosectomy immediately after drainage yields favorable outcomes.^2^ However, the necrotic collections in that study were of limited size, raising questions as to whether these findings can be generalized to patients with more complicated WON. We hypothesize that an accelerated treatment algorithm for patients with large WON could reduce hospital length of stay (LOS) and decrease the incidence of major complications.

**Aims & Methods**: This single‐center, open‐label, randomized controlled superiority trial enrolled patients with WON with a diameter >15 cm, who were assigned to either an accelerated or conventional step‐up treatment algorithm. Both groups underwent transgastric drainage with a 20 mm lumen‐apposing metal stent. In the accelerated group, necrosectomy was performed during the index procedure and continued as needed until the WON cavity was cleared of necrotic tissue. In the step‐up group, subsequent endoscopic necrosectomy or additional drainage was only performed if clinical improvement was not observed. The primary endpoint was a composite of death during treatment, major complications (as defined by the TENSION protocol^3^), and LOS exceeding 58 days from the index procedure. The endpoint of LOS exceeding 58 days was chosen based on a recent trial performed at our center, which found a median LOS in comparable patients to be 58 days.^4^ Secondary outcomes included mortality, major complications, and LOS. To ensure the safety of the accelerated approach, an interim analysis was performed after treatment of 25 patients according to protocol (ClinicalTrials.gov NCT05601687).

**Results**: A total of 25 patients were included in this interim analysis: 12 in the accelerated group and 13 in the step‐up group. The groups were comparable in terms of gender distribution, age, Charlson Comorbidity score, etiology of acute pancreatitis, indication for treatment, and WON size, with an overall median diameter of 21.7 cm (interquartile range [IQR] 18.0–25.0), defined as the largest measured diameter of the WON on cross‐sectional imaging. The primary composite outcome occurred in 2 of 12 patients (16.6%) in the accelerated group and in 9 of 13 patients (69.3%) in the step‐up group (odds ratio [OR] 0.089; *p* = 0.015). One patient in the step‐up group died because of hemorrhage 14 days after index procedure. Major complications were significantly lower in the accelerated group (8.3% vs. 53.8%; OR 0.078; *p* = 0.030). In the step‐up group, the first encountered major complication occurred a median of 38 days [IQR 24–42] after initial drainage. The median LOS was significantly shorter in the accelerated group (32.5 days [IQR 17.5–38.2] vs. 68.5 days [IQR 35.5–97.8], *p* = 0.039).

**Conclusion**: An accelerated treatment algorithm seems to reduce both the rate of major complications and the LOS compared to a conventional step‐up approach, suggesting that a more aggressive treatment approach is preferable in patients with large symptomatic WON.

**References**

1. Ebrahim M, Werge MP, Hadi A, Lahchich M, Nagras ZG, Lauritsen ML, et al. Clinical outcomes following endoscopic or video‐assisted retroperitoneal management of acute pancreatitis with large (>15 cm) walled‐off pancreatic necrosis: Retrospective, single tertiary center cohort study. *Dig Endosc*. 2022;34(6):1245–1252. 10.1111/den.14295
2. Bang JY, Lakhtakia S, Thakkar S, Buxbaum JL, Waxman I, Sutton B, et al. Upfront endoscopic necrosectomy or step‐up endoscopic approach for infected necrotising pancreatitis (DESTIN): a single‐blinded, multicentre, randomised trial. *Lancet Gastroenterol Hepatol*. 2024;9(1):22–33. 10.1016/S2468‐1253(2300331‐X)
3. van Brunschot S, van Grinsven J, van Santvoort HC, Bakker OJ, Besselink MG, Boermeester MA, et al. Endoscopic or surgical step‐up approach for infected necrotising pancreatitis: a multicentre randomised trial. *Lancet*. 2018;391(10115):51–58. 10.1016/S0140‐6736(1732404‐2)
4. Karstensen JG, Novovic S, Hansen EF, Jensen AB, Jorgensen HL, Lauritsen ML, et al. EUS‐guided drainage of large walled‐off pancreatic necroses using plastic versus lumen‐apposing metal stents: a single‐centre randomised controlled trial. *Gut*. 2023;72(6):1167–1173. 10.1136/gutjnl‐2022‐328225

**Disclosure**: Nothing to disclose.

## LB17 | DIGITAL CONFOCAL MICROSCOPY FOR REAL‐TIME DIAGNOSIS OF PANCREATIC SOLID LESION: A MULTICENTER STUDY

S. Stigliano ^1^, G. Rossi^2^, C. Taffon^3^, G. Soy^4^, P. G. Arcidiacono^5^, C. Doglioni^6^, A. Crescenzi^7^, A. Gines^8^, F. M. Di Matteo^1^

^1^ *Therapeutic Endoscopy Unit, Fondazione Policlinico Universitario Campus Bio‐Medico, Roma, Italy*, ^2^ *Pancreato‐Biliary Endoscopy and Endosonography Division, Pancreas Translational and Clinical Research Center, San Raffaele Scientific Institute IRCCS; Vita S, Milano, Italy*, ^3^ *Fondazione Policlinico Universitario Campus Bio‐Medico, Rome, Italy*, ^4^ *Clinic Hospital of Barcelona, Barcelona, Spain*, ^5^ *PancreatoBiliary Endoscopy and Endosonography Division, Pancreas Translational and Clinical Research Center, Ospedale San Raffaele, Milan, Italy*, ^6^ *IRCCS San Raffaele Scientific Institute, Milan, Italy*, ^7^ *La Sapienza, Roma, Italy*, ^8^ *Department of Gastroenterology, Hospital Clínic de Barcelona, Barcelona, Spain*

**Contact E‐Mail Address**: s.stigliano@policlinicocampus.it

**Introduction**: The feasibility of Fluorescence Confocal laser microscopy (FCM) with MAVIG VivaScope® in EUS‐FNB samples of pancreatic solid lesion has been assessed, showing good concordance with the final histological evaluation.

**Aims & Methods**: We aim to evaluate the accuracy of FCM in predicting the diagnosis of pancreatic solid lesions EUS‐TA samples and its agreement with the final histological diagnosis.

Secondary aim: To assess the possible association between type and size of needles and the histological diagnosis obtainment.

Multicenter prospective study involving three high level European Hospitals. Obtained samples have been evaluated at FCM and classified as “Inadequate” or “Adequate” (Benign; Suspicious; Malignant). The type and size of needle used and the technique used were recorded.

**Results**: From April 2023 to August 2023, 80 patients were enrolled (50% male; mean age 66 ± 14 years old). In 78.5% FNB needle was used. In 66.3% procedures a 22G needle was used. The sample obtained was defined Adequate at VivaScope® in 96.3% cases. The diagnosis at VivaScope® was categorized in 5.2% benign, 11.7% suspicious and 83.1% malignant. The use of FNB needle was not associated with a higher rate of diagnostic samples at Vivascope evaluation (88.8% vs. 100% *p* 0.33) and neither the needle size (19 G 100% vs. 22 G 88.6% vs. 25 G 96.2 *p* 0.51). There was no difference in term of sampling technique and the rate of diagnostic sample at Vivascope evaluation (93.9% vs. 78.5% *p* 0.09).

The VivaScope®'s Sensitivity was 97.33% (95% CI 90.7%–99.7%) the Specificity was 80.0% (95% CI 28.4%–99.5%) and the overall Accuracy was 96.3% (95% CI 89%–99.2%).

Overall, there was moderate concordance between the Vivascope diagnosis and the final histological diagnosis (kappa Cohen's coefficient 0.58).

**Conclusion**: The FCM can provide fast information about sample adequacy and diagnosis of EUS TA with high accuracy. The use of FCM could eliminate the differences between needle type and size linked with diagnostic sample obtainment.

**Disclosure**: Nothing to disclose.

## LB18 | PULMONARY IMPAIRMENT IN CHRONIC PANCREATITIS: A LONGITUDINAL AND COMPARATIVE STUDY WITH HEALTHY CONTROLS (RESPPANC STUDY)

A. Hadi ^1^, M. P. Werge^1^, J. G. Karstensen^1,2^, E. Frausing Hansen^3^, L. L. Gluud^1,2^, S. Novovic^1,2^

^1^ *Gastro Unit, Pancreatitis Centre East, Copenhagen University Hospital ‐ Amager and Hvidovre, Hvidovre, Denmark*, ^2^ *Department of Clinical Medicine, Faculty of Health and Medical Sciences, University of Copenhagen, Copenhagen, Denmark*, ^3^ *Department of Respiratory Medicine, Copenhagen University Hospital ‐ Amager and Hvidovre, Hvidovre, Denmark*

**Contact E‐Mail Address**: amerhadi89@gmail.com

**Introduction**: Chronic pancreatitis (CP) is an irreversible fibro‐inflammatory syndrome leading to the deterioration of pancreatic function. CP can progress from acute pancreatitis (AP) although the pathophysiology is not fully understood.^1^ Pulmonary dysfunction is the primary extra‐pancreatic complication during AP, persisting for extended periods.^2^ The fibro‐inflammatory response during CP is milder than in AP but is often long‐lasting and repetitive; hence, the same fibro‐inflammatory process may induce chronic changes in lung tissue.^3^

**Aims & Methods**: The objective of this prospective cohort study was to examine the pulmonary function in CP patients over time and compare with healthy controls. The primary endpoints were change in percent predicted (% pred) diffusion capacity for carbon monoxide (DLCO) and alveolar volume (VA). Patients were enrolled from the outpatient clinic of a tertiary referral center. Inclusion criteria were: confirmed CP according to the M‐ANNHEIM criteria,^4^ no current tobacco use and no confirmed pulmonary conditions excluding chronic obstructive pulmonary disease (COPD). Additionally, hospital admission, infection or use of antibiotics must not be present within 2 months preceding the assessments at baseline, year 1, and year 3.

Each visit included disease staging, patient characteristics, questionnaires, pulmonary function‐ and diffusion tests, evaluation of strength including respiratory muscles and chest X‐ray. Healthy controls underwent a single evaluation without imaging, according to protocol (ClinicalTrials.gov: NCT03850977).

**Results**: From 2018 to 2023, 55 patients with CP were included with follow‐up of 93% (*N*_1_ = 51) at year 1% and 80% (*N*_3_ = 44) at year 3. Eighteen healthy controls were matched in age, gender and smoking status; however, the CP cohort had a higher former misuse of alcohol (22% vs. 0%, *p* < 0.01). As expected, 11% (*N* = 6) had COPD.

Compared to healthy controls, CP patients had a lower median % pred DLCO (85 [67–93] vs. 91 [86–95], *p* < 0.01), VA (88 [80–95] vs. 94 [91–98], *p* = 0.02), forced expiratory volume in 1 s (FEV1) (93 [83–102] vs. 104 [96–108], *p* = 0.01), forced vital capacity (FVC) (100 [90–108] vs. 106 [98–113], *p* = 0.05) and total lung capacity (TLC) (88 [80–95] vs. 95 [92–98], *p* = 0.01). No differences were observed in DLCO/VA (*p* = 0.61), FEV1/FVC (*p* = 0.12) and peak expiratory flow (PEF) (*p* = 0.25).

In CP patients, pulmonary function was not correlated to M‐ANNHEIM stage or self‐reported dyspnea. Compared to non‐smokers, ex‐smokers with CP had a significantly lower % pred DLCO/VA (*p* = 0.04) and lower FEV1/FVC (*p* = 0.006). Other parameters showed no difference. Patients with alcohol as etiology had a lower % pred DLCO (*p* = 0.04) and DLCO/VA (*p* = 0.0095).

During follow‐up, CP patients experienced a significant decrease in pulmonary function, see table.

**Table:** 

	Baseline (mean)	Change at year 1	*p* value	Change at year 3	*p* value
DLCO*	80.1 (75.3–84.9)	−1.9 (−3.6 to −0.2)	0.03	−3.12 (−5.8 to −0.5)	0.02
VA*	87.7 (84.4–91.0)	−1.2 (−3.0 to 0.53)	0.17	−2.9 (−5.1 to −0.8)	0.009
DLCO/VA*	91.8 (84.5–97.1)	−1.6 (−4.2 to 1.0)	0.22	−0.79 (−3.6 to 2.0	0.57
FEV1*	94.0 (89.0–98.9)	−2.83 (−5.0 to −0.7)	0.01	−2.9 (−5.5 to −0.4)	0.03
FVC*	99.3 (94.8–103.7)	−2.4 (−4.4 to −0.3)	0.02	−1.3 (−3.9 to 1.4)	0.34
FEV1/FVC**	0.77 (0.74–0.79)	−0.012 (−0.03 to 0)	0.07	−0.03 (−0.04 to −0.01)	0.0003
TLC*	88.0 (84.8–91.2)	−0.86 (−2.4 to 0.7)	0.28	−3.0 (−5.2 to 0.89)	0.007

*% pred on age, gender, ethnicity, height and weight. **Ratio of absolute values.

**Conclusion**: Patients with CP have decreased pulmonary function compared to healthy controls. Additionally, the decline in pulmonary function is more rapid. Smoking cannot explain the findings, indicating the fibro‐inflammatory process associated with CP may have pulmonary consequences.

**References**

1. Vege SS, Chari ST. Chronic Pancreatitis. Solomon CG, ed. *N Engl J Med*. 2022;386(9):869–878. 10.1056/NEJMCP1809396
2. Chelliah T, Werge M, Merc AI, Bisgaard T, Hansen EF, Hansen EF, et al. Pulmonary dysfunction due to combination of extra‐pulmonary causes and alveolar damage is present from first the day of hospital admission in the early phase of acute pancreatitis. *Pancreatology*. 2019;19(4):519–523. 10.1016/J.PAN.2019.04.009
3. Masoero G, Spinaci S, Arossa W, Andriulli A, Gaia E, De Pretis G, et al. Pulmonary involvement in chronic pancreatitis. *Dig Dis Sci*. 1984;29(10):896–901. 10.1007/BF01312477
4. Schneider A, Löhr JM, Singer MV. The M‐ANNHEIM classification of chronic pancreatitis: Introduction of a unifying classification system based on a review of previous classifications of the disease. *J Gastroenterol*. 2007;42(2):101–119. 10.1007/s00535‐006‐1945‐4

**Disclosure**: Nothing to disclose.

## LB19 | REDUCTIONS IN THE NAFLD FIBROSIS SCORE FROM THE ZENITH‐CKD TRIAL SUPPORT EXPLORATION OF ZIBOTENTAN DAPAGLIFLOZIN COMBINATION IN CIRRHOSIS

P. Ambery ^1^, P. Greasley^2^, E. Wijkmark^3^, K. Persson^3^, J. Oscarsson^4^, K. Fiorino^5^, S. Ravikiran^6^, H. J. L. Heerspink^7,8^

^1^ *Clinical Late Development, Cardiovascular, Renal and Metabolism, BioPharmaceuticals R&D, AstraZeneca, Gothenburg, Sweden*, ^2^ *Research and Early Development, Cardiovascular, Renal and Metabolism, BioPharmaceuticals R&D, AstraZeneca, Gothenburg, Sweden*, ^3^ *Biometrics Late Development, Cardiovascular, Renal and Metabolism, BioPharmaceuticals R&D, AstraZeneca, Gothenburg, Sweden*, ^4^ *Late‐Stage Clinical Development, Cardiovascular, Renal, and Metabolism, BioPharmaceuticals R&D, AstraZeneca, Gothenburg, Sweden*, ^5^ *Clinical Development, Cardiovascular, Renal, and Metabolism, BioPharmaceuticals R&D, AstraZeneca, Gaithersburg, Maryland, USA*, ^6^ *Biometrics Late‐Stage Development, Cardiovascular, Renal, and Metabolism, BioPharmaceuticals R&D, AstraZeneca, Gaithersburg, Maryland, USA*, ^7^ *Department of Clinical Pharmacy and Pharmacology, University of Groningen, University Medical Center Groningen, Groningen, Netherlands*, ^8^ *The George Institute for Global Health, University of New South Wales, Sydney, New South Wales, Australia*

**Contact E‐Mail Address**: phil.ambery@astrazeneca.com

**Introduction**: Positive effects on markers of metabolic dysfunction–associated steatotic liver disease (MASLD) have already been demonstrated for sodium–glucose cotransporter‐2 (SGLT2) inhibitors. Pre‐clinical evidence from rodent models also suggests MASLD benefits of endothelin‐A receptor antagonism. Zibotentan, the most selective endothelin A receptor antagonist, is currently under clinical investigation in combination with dapagliflozin for treatment of chronic kidney disease (CKD) and for liver cirrhosis. Advanced CKD is also recognized to occur concomitantly with liver cirrhosis.

**Aims & Methods**: The aim of this exploratory analysis was to evaluate the effect of zibotentan combined with dapagliflozin and dapagliflozin alone on a non‐invasive measure of MASLD in patients with CKD recruited into the ZENITH‐CKD phase 2b study.

447 patients in the ZENITH‐CKD study were treated for 12 weeks with either zibotentan 0.25 mg in combination with dapagliflozin 10 mg (*n* = 91), or zibotentan 1.5 mg in combination with dapagliflozin 10 mg (*n* = 179), or dapagliflozin 10 mg alone (active control) (*n* = 177). The primary outcome was urinary albumin creatinine ratio at week 12. The presented results are from patients with non‐alcoholic fatty liver disease fibrosis score (NFS) > 0.675, indicating high risk for advanced fibrosis and included 39 patients treated with combination therapy with either dose of zibotentan, and 27 patients treated with dapagliflozin alone.

**Results**:

**Table:** 

	Zibotentan 0.25 mg/dapagliflozin 10 mg combination group	Zibotentan 1.5 mg/dapagliflozin 10 mg combination group	Zibotentan/dapagliflozin combination groups (pooled)	Dapagliflozin 10 mg alone
Baseline NFS				
*n*	10	29	39	27
Median (Q1, Q3)	1.03 (0.93, 1.23)	1.37 (0.83, 1.77)	1.12 (0.83, 1.58)	1.21 (0.99, 1.62)
NFS reduction at week 12 from baseline				
*n*	6	24	30	20
Median (Q1, Q3)	0.56 (0.40, 0.69)	0.09 (−0.16, 0.29)	0.13 (−0.08, 0.50)	0.26 (−0.30, 0.54)

**Conclusion**: A reduction in NFS was observed in this small exploratory analysis of patients with CKD and high risk for advanced liver fibrosis across all three active arms of the study. These data support further exploration of the effects of zibotentan and dapagliflozin in patients with liver cirrhosis as part of the ongoing ZEAL part B study. Further data from both the CKD and liver study programs are awaited.

**Disclosure**: PA, PG, EW, KP, JO, KF and SR are employees of, and hold stakes in, AstraZeneca. HJLH has received funding from AstraZeneca, Bayer, Boehringer Ingelheim, Janssen, and Novartis. HJLH has received consulting fees from Alexion, AstraZeneca, Bayer, Boehringer Ingelheim, CSL Behring, Dimerix, Eli Lilly, Janssen, Novartis, Novo Nordisk, Roche, Travere Therapeutics and Vifor Pharma.

## LB20 | L‐CARNITINE EFFICACY FOR MANAGEMENT OF NON‐ALCOHOLIC FATTY LIVER DISEASE: A MULTI CENTRIC RANDOMIZED CLINICAL TRIAL

P. Alavinejad ^1^, S. Zaky^2^, N. S. Behl^3,4^, A. Mohammed Mosaad^2^, M. Gamal Abderabu^5^, n. elgazzar^6^, E. Hussein Altaweel^2^, M. Ahmed Haridy^2^, E. Ghoneem^7,8^, A. Hassan^2^, E. Lak^1^, M. Yousry Soliman^9^, M. Hussien Ahmed^10^, M. Arshadzadeh^11^, A. Hormati^12^, A. M. Mahros^13^, V. H. Dao^14^, M. J. Rezaei^1^, S. A. Lashen^15^, R. Ibrahim Salma^16^, A. Nejad salami^1^, A. Eslami^1^, Q.T. Tran^17^, M. Hagag^2^

^1^ *GI Department, Ahvaz Jundishapur University of Medical Sciences, Ahvaz, Iran*, ^2^ *Hapatogastroenterology, Al Azhar University, Cairo, Egypt*, ^3^ *S.M.S Medical College and Hospitals, Jaipur, Rajasthan*, ^4^ *Department of Gastroentrology, Ludhiana, India*, ^5^ *Alexandria Faculty of Medicine, Alexandria, Egypt*, ^6^ *Tanta Faculty of Medicine, Tropical Medicine, Tanta, Egypt*, ^7^ *Faculty of Medicine Mansoura University/Egyptian Liver Hospital, Mansoura, Egypt*, ^8^ *Hepatogastroenterology, Mansoura‐Dakahleya, Egypt*, ^9^ *Hepatogastroenterology and Infectious Diseases, Al‐Azhar University‐Faculty of Medicine, Tanta, Egypt*, ^10^ *Tropical Medicine, Gastroentrology, Kafrelsheikh Unversity, Kafrelsheikh, Egypt*, ^11^ *Tehran University of Medical Sciences, Tehran, Iran*, ^12^ *Gastroenterology and Hepatology Disease Research Center, Qom University of Medical Sciences, Qom, Iran*, ^13^ *Gastroentrology, Hepatology and Infectious Disease, Kafr El Sheikh University, Kafr Elsheikh, Egypt*, ^14^ *Internal Medicine Faculty, Hanoi Medical University, Hanoi, Vietnam*, ^15^ *Hepatogastroenterology Division, Internal Medicine, Alexandria University, Faculty of Medicine, Alexandria, Egypt*, ^16^ *Zagazig University, Zagazig, Egypt*, ^17^ *Internal Medicine A, Hue University, Hue, Vietnam*

**Contact E‐Mail Address**: Pezhmanalavinejad@gmail.com

**Introduction**: Nonalcoholic fatty liver disease (NAFLD), the hepatic manifestation of metabolic syndrome, is the leading cause of liver failure and transplantation worldwide. The primary treatment for this disease is lifestyle modification and weight reduction. This study aims to evaluate the impact of L‐carnitine, which plays a crucial role in lipid metabolism and beta‐oxidation of long‐chain fatty acids in mitochondria, on improving non‐alcoholic fatty liver disease.

**Aims & Methods**: During a 6 month period, NAFLD patients from 5 referral centers in 3 countries randomly divided into two groups: L‐carnitine supplementation group (group A) and placebo group (Group B). Firstly, the metabolic profile of participants determined and recorded. Then, participants in the intervention group received L‐carnitine (1000 mg twice a day) for 3 months and in group B, the participant just received advisement for lifestyle modification. After 12 weeks, they reevaluated for biophysical profile and hepatic fat status by using ultrasound and these parameters compared between two groups.

**Results**: Overall, 393 NAFLD cases included (216 males [55.2%], 205 cases in group A & 188 cases in group B, average age 44.8 years). Participants were originally from 11 countries and racially 76.7% of them were Middle Eastern/North African. The most common co morbidity were hyperlipidemia (47.6%), hypertension (40.5%) and diabetes mellitus (31.6%). After 12 weeks of intervention, average serum level of ALT in group A (L carnitine) decreased from 65.6 to 40.9, while in group B, changed from 62.8 to 50.7 (*p* = 0.0029). Average serum level of AST in group A decreased from 50.9 to 35.7 and in group B changed from 54.7 to 44.85 (*p* = 0.046). During ultra‐sonographic evaluation, the percentile of those who diagnoses as NAFLD grade 3 in group A, from 37.9% before intervention decreased to 12.6% while in control group (B), changed from 36.6% to 22.7% (*p* < 0.05).

**Conclusion**: L carnitine supplementation is effective in management of NAFLD patients and can improve their biophysical profile including liver transaminases and ultra‐sonographic grade of fatty liver.

**Disclosure**: Nothing to disclose.

